# Florida Current transport observations reveal four decades of steady state

**DOI:** 10.1038/s41467-024-51879-5

**Published:** 2024-09-05

**Authors:** Denis L. Volkov, Ryan H. Smith, Rigoberto F. Garcia, David A. Smeed, Ben I. Moat, William E. Johns, Molly O. Baringer

**Affiliations:** 1 Cooperative Institute for Marine and Atmospheric Studies, University of Miami, Miami, FL USA; 2 NOAA Atlantic Oceanographic and Meteorological Laboratory, Miami, FL USA; 3 National Oceanography Centre, Southampton, UK; 4 Rosenstiel School of Marine, Atmospheric, and Earth Science, University of Miami, Miami, FL USA

**Keywords:** Physical oceanography, Physical oceanography

## Abstract

The potential weakening of the Atlantic Meridional Overturning Circulation (AMOC) in response to anthropogenic forcing, suggested by climate models, is at the forefront of scientific debate. A key AMOC component, the Florida Current (FC), has been measured using submarine cables between Florida and the Bahamas at 27°N nearly continuously since 1982. A decrease in the FC strength could be indicative of the AMOC weakening. Here, we reassess motion-induced voltages measured on a submarine cable and reevaluate the overall trend in the inferred FC transport. We find that the cable record beginning in 2000 requires a correction for the secular change in the geomagnetic field. This correction removes a spurious trend in the record, revealing that the FC has remained remarkably stable. The recomputed AMOC estimates at ~26.5°N result in a significantly weaker negative trend than that which is apparent in the AMOC time series obtained with the uncorrected FC transports.

## Abstract (web summary)

The study shows that the Florida Current, an important contributor to the Atlantic Meridional Overturning Circulation, has not declined as previously suggested, but remained steady over the past 40-years of observations.

## Introduction

Originating as waters from the Gulf of Mexico Loop Current that enter the western Straits of Florida, the Florida Current (FC) is constrained by the relatively shallow bathymetry ( < 800-m depth) between the Florida Peninsula, Cuba, and the Bahamas before entering the open North Atlantic and becoming the Gulf Stream. This strong, upper-ocean flow, where the surface currents can reach velocities of 2 m s^−1^^1^, represents the beginning of and the swiftest part of the Gulf Stream System. The FC carries approximately 32 Sv (1 Sv = 10^6^ m^3^ s^−1^) of warm and salty tropical waters northward and plays an important role in the regional and global climate systems^2,3^. Bolstered by western boundary intensification, the FC provides most of the northward volume and heat transport in the subtropical North Atlantic and accounts for the bulk of both the upper limb of the Atlantic Meridional Overturning Circulation (AMOC) and the western boundary component of the subtropical gyre circulation. Therefore, observing and investigating the variability of the FC is of particular importance for detecting and better understanding changes in the Earth’s climate system, as well as for interpreting regional variations in weather, sea level, and ecosystems.

**Fig. 1:**
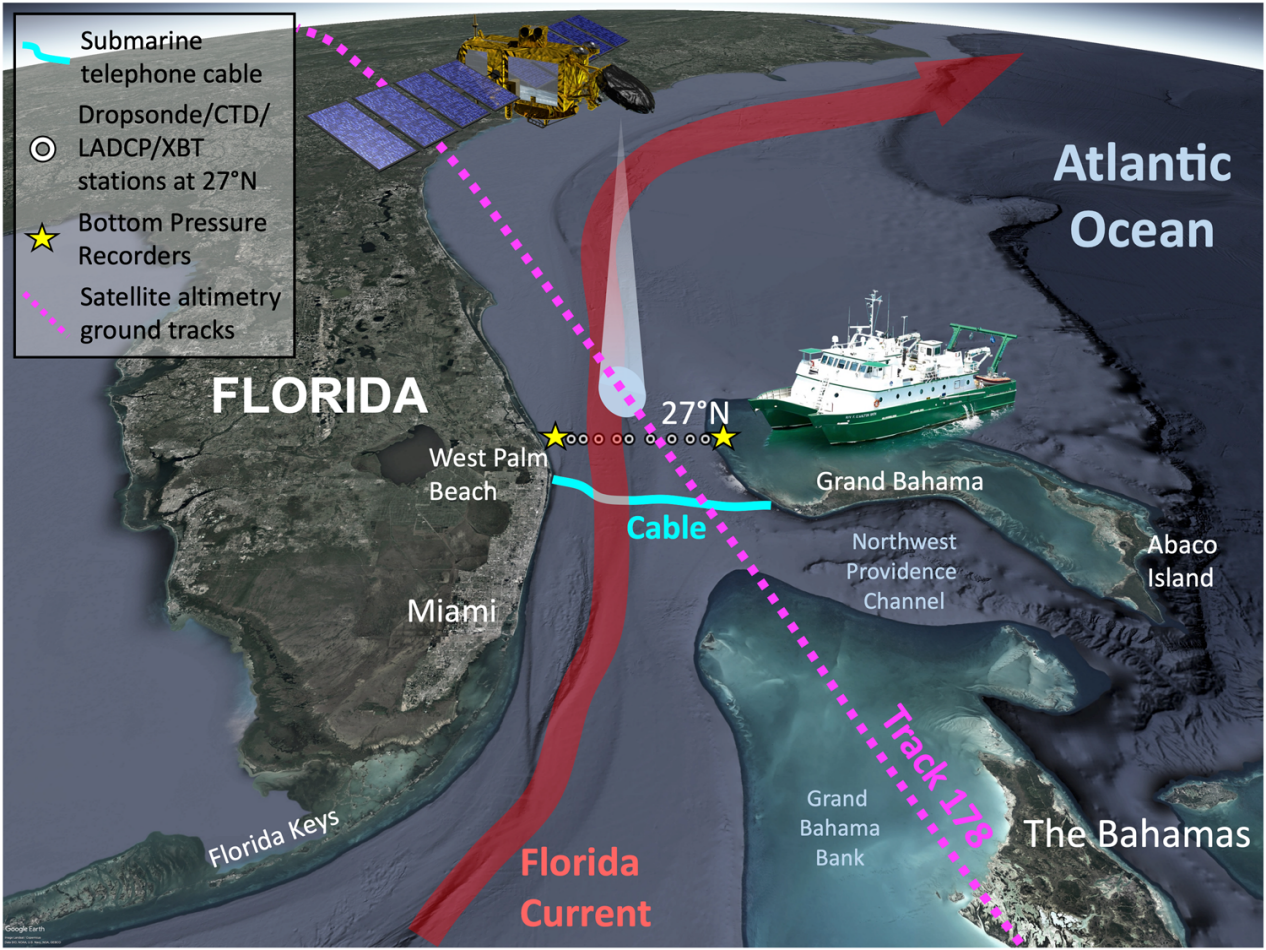
The Western Boundary Time Series observing network in the Straits of Florida. The network consists of the submarine telecommunications cable between West Palm Beach and Grand Bahama Island (cyan curve), ship sections across the Florida Current (FC) at 27°N with in situ measurements at nine stations (white circles), two bottom pressure gauges on both sides of the FC at 27°N (yellow stars), and along-track satellite altimetry measurements (magenta dotted line). CTD Conductivity-Temperature-Depth, LADCP Lowered Acoustic Doppler Current Profiler, XBT expendable bathythermograph.

A unique, sustained observing system in the Florida Straits, consisting of voltage measurements recorded from a submarine telecommunication cable installed between Florida and Grand Bahama Island, paired with regular ship surveys in the Florida Straits at 27°N (Fig. 1), was established in 1982^4–8^. Voltage perturbations induced on the cable by the varying FC flow through the Earth’s magnetic field (EMF; henceforth, used interchangeably with the geomagnetic field) are calibrated and processed into volume transport estimates using transports calculated from ship section data, collected with Pegasus floats in 1982–1988^5,6,9^, with free-falling dropsonde floats since 1991^10^, and with Lowered and Shipboard Acoustic Doppler Current Profilers (LADCP and SADCP) since 2001^8^. Since 2000, this observing system has been maintained by the National Oceanic and Atmospheric Administration’s (NOAA) Atlantic Oceanographic and Meteorological Laboratory (AOML), in Miami, Florida, as part of the Western Boundary Time Series (WBTS) program. As of now, the cable measurements have provided nearly 40 years of quasi-continuous, daily estimates of the FC volume transport. Beginning in 2004, WBTS partnered with the Rapid Climate Change (RAPID) and Meridional Overturning Circulation and Heatflux Array (MOCHA) programs, linking the Florida Straits WBTS cable recording system with RAPID/MOCHA tall moorings deployed between the Bahamas and Africa at about 26.5°N. The resulting RAPID-MOCHA-WBTS array (henceforth RAPID array) is the oldest trans-basin AMOC observing array in existence (e.g.^11^,).

The AMOC is the principal driver for oceanic meridional heat transport in the subtropical North Atlantic^12^. Most climate models that contributed simulations to the Coupled Model Intercomparison Project Phase 6 (CMIP6) suggest a decline of the AMOC by 34–45% of its present-day strength towards the end of the 21^st^ century^13^. A variety of published proxy records indicate that the AMOC may already be slowing down on a centennial time scale^14–17^. Some proxy-based conclusions, however, have lately been criticized, as being based only on a subset of proxy records^18^. The ability of temperature-based indices to represent the AMOC has also been questioned^19^. In addition, it has recently been shown that the observed reduction in sea surface temperatures in the subpolar North Atlantic (also known as the warming hole), often assumed to be an indication of the AMOC slowdown, could largely be driven by the atmosphere alone without any changes in ocean circulation^20^. Direct observations at the RAPID array have shown that the AMOC was in a state of reduced overturning in 2008 and 2016 as compared to the 2004–2008 period^21^. On the other hand, the AMOC transport time series shows a small, but not statistically significant, increase between 2009 and 2018^22^. Similarly, direct measurements obtained from repeat sections across the Gulf Stream, between New Jersey and Bermuda (beginning in 1992), do not support suggestions that the AMOC has started slowing down substantially^23–25^, and no significant weakening of the AMOC has been detected in reconstructions based on sparse hydrographic section data^26–28^.

It is possible that the AMOC time series derived from direct measurements are still too short, and primarily showcase interannual-to-decadal variations^29,30^. If the AMOC is indeed slowing down on a centennial timescale, its upper branch, primarily composed of the FC and the Gulf Stream in the subtropical North Atlantic, may also be weakening^31^, although the trends downstream and upstream of Cape Hatteras can be different^32^. The four-decade long FC transport time series inferred from cable measurements at 27°N represents the longest observational record of a key AMOC component. This record has exhibited a small negative trend of about −0.3 Sv per decade. This trend has been monitored and documented in several State of the Climate reports published as supplements to the Bulletin of the American Meteorological Society (e.g.^33–38^,). Depending on the year of publication, the trend has been deemed either insignificant or marginally significant, therefore, not attracting particular attention. Several recent studies, however, have used the overall trend in the FC transport to conjecture about the possible centennial decline of the Gulf Stream and the AMOC^39–41^.

The objective of this study is to reassess the record-long change in the FC strength over the four decades of observations. Specifically, we reconsider the processing of voltages measured on the submarine cable that are used to derive the FC volume transport time series. Most importantly, we apply a correction for the secular change in the EMF to the voltage data to derive corrected FC volume transports since 2000. This correction was accounted for in the earlier (1982–1998) part of the cable record^7^ but neglected later due to a change in data processing more heavily reliant upon calibration with ship section data. We show that with the increased length of the cable record, explicit consideration of local geomagnetic field variations has become necessary, especially when studying processes on decadal and longer time scales. The analysis of the corrected time series presented herein challenges the previously made assertions on the statistically significant decline of the FC at 27°N^39,40,42^. Finally, we discuss the implications of the corrected FC time series on the AMOC estimates at the RAPID array.

## Results

### Long-term changes in the Florida current strength

**Fig. 2:**
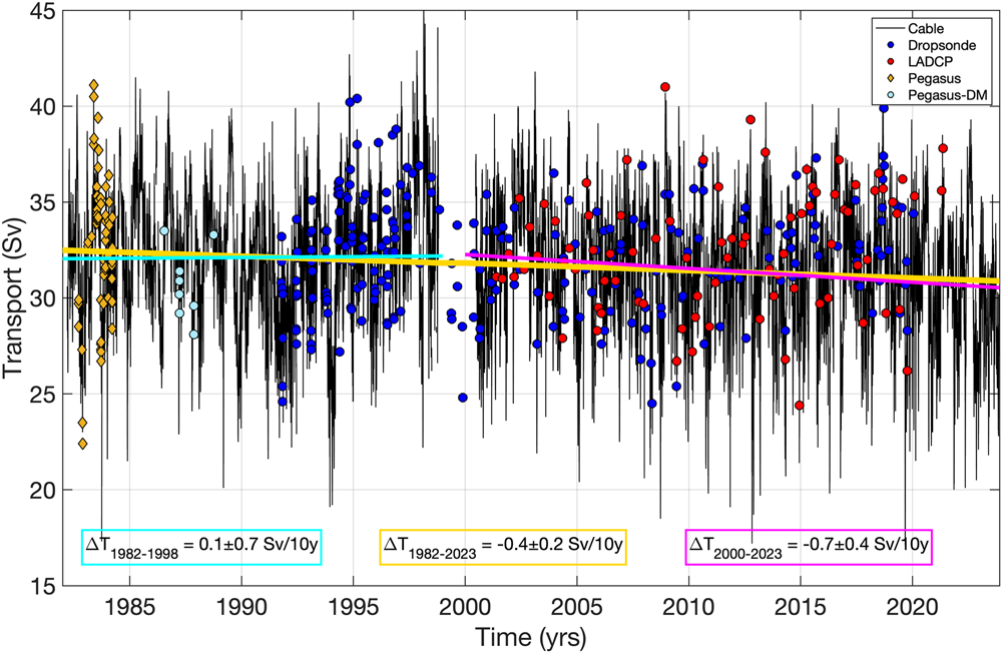
The Florida Current volume transport. Daily transport estimates from the cable record (black; prior to corrections applied in this study); estimates from the Pegasus (orange diamonds) and Pegasus in dropsonde mode (Pegasus-DM; light blue circles) sections; estimates from the dropsonde sections (blue circles); and estimates from the Lowered Acoustic Doppler Current Profiler (LADCP) sections (red circles). The linear trends for 1982–2023, 1982–1998, and 2000–2023 periods are shown by the orange, cyan, and magenta lines, respectively.

The daily FC volume transports inferred from cable voltages and estimated from ship section data at 27°N from 1982 to 2023, prior to corrections applied in this study, are presented in Fig. 2. The cable record is nearly continuous except several gaps that occurred due to operational issues and instrument failures (“Methods”). The cable transport shows a time mean of 31.7 Sv and a standard deviation of 3.4 Sv. The ship sections yield a time mean of 32.3 Sv and a standard deviation of 3.1 Sv. The FC transport variability is dominated by subseasonal (periods less than a year) signals, while the interannual-to-decadal variability is weak, with a standard deviation of less than 1 Sv^3,43,44^. The 1982–2023 linear trend of the FC volume transport from the cable was −0.4 ± 0.2 Sv per decade (the uncertainty indicates the 95% confidence interval; see Methods). Note that this trend is significant and greater than the trend of about −0.3 Sv per decade reported in earlier studies^33–36,39,40^. The trend became significant relative to the noise as the time series length increased and due to low transports in the most recent 2–3 years of observations now included in the record. During these recent years, the FC transport was about 1.5 Sv lower than the record average^45^.

It is important to note that the negative trend in the time series is only evident in the data after 2000, when cable measurements resumed following a 17-month long (October 1998—March 2000) cessation (“Methods”). Indeed, the 1982–1998 trend of 0.1 ± 0.7 Sv per decade is very small and insignificant, while the 2000–2023 period yields a statistically significant trend of −0.7 ± 0.4 Sv per decade. This observation is consistent with^39^, where it was reported that a significant decline in Gulf Stream transport has only become detectable during the past decade. We note, however, that since the decline can be traced back to the resumption of cable measurements in 2000, it is possible that the observed decline in the FC transport could be due to a change in the data processing procedure (see “Methods” for details) rather than the real oceanic signal.

**Fig. 3:**
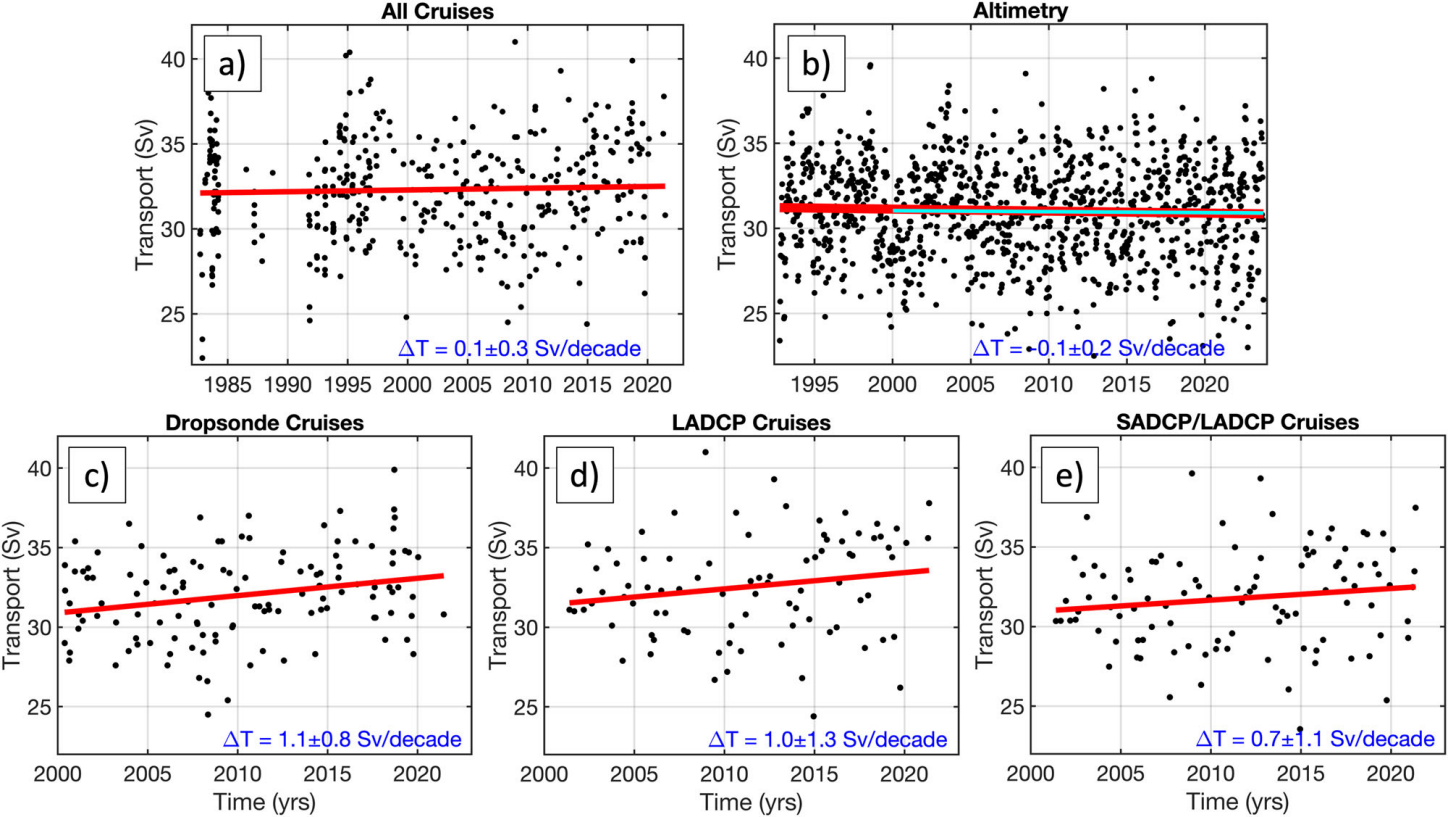
The Florida Current volume transport estimates from ship sections and satellite altimetry. Linear trends (red lines) of the Florida Current volume transports estimates (black dots) from (**a**) all ship sections, (**b**) satellite altimetry, (**c**) dropsonde sections, (**d**) lowered acoustic doppler current profiler (LADCP) sections, and (**e**) the combination of Shipboard Acoustic Doppler Current Profiler (SADCP) and LADCP sections. The cyan line in (**b**) shows the linear trend in 2000–2023.

To validate the trends in the cable data, we also estimate the linear trends in the transports estimated from ship section data and from cross-stream satellite altimetry measurements (“Methods”). The trend estimates for different observing platforms and time intervals are presented in Table 1 and in Fig. 3. The estimates from all ship sections combined in 1982–2023 show a small and statistically insignificant trend of 0.1 ± 0.3 Sv per decade (Fig. 3a). The same trend (0.1 ± 0.4 Sv per decade) is obtained from all ship sections in 1993–2023. Within the uncertainty bounds, this trend agrees with the altimetry-derived trend of −0.1 ± 0.2 Sv per decade for the 1993–2023 period (Fig. 3b). It is interesting to note that the altimetry-derived transports for the 2000–2023 period show the same, statistically insignificant negative trend of −0.1 ± 0.3 Sv per decade (cyan line in Fig. 3b). On the other hand, the transports estimated from the dropsonde, LADCP, and the combination of LADCP and SADCP (LADCP/SADCP) sections in 2000–2023 exhibit positive trends of about 1 Sv per decade, although only the trend obtained from the dropsonde sections (1.1 ± 0.8 Sv per decade) is found to be significantly greater than zero (Fig. 3c). Both the LADCP and the LADCP/SADCP sections yield insignificant trends of 1.0 ± 1.3 Sv per decade and 0.7 ± 1.1 Sv per decade, respectively (Fig. 3d, e). It should be noted that ship section data is relatively sparse and is biased towards good weather days as no calibration cruises are carried out in adverse weather conditions.

**Table 1:** Linear trends of the Florida Current volume transport

Time period	1982–2023	1982–1998	1993–2023	2000–2023
Cable	−0.4 ± 0.2	0.1 ± 0.7	− 0.5 ± 0.3	− 0.7 ± 0.4
Cable^EMF^	− 0.1 ± 0.2	0.1 ± 0.7	− 0.1 ± 0.3	0.0 ± 0.3
All cruises	0.1 ± 0.3	0.4 ± 0.9	0.1 ± 0.4	1.1 ± 0.7
Dropsonde			− 0.1 ± 0.5	1.1 ± 0.8
LADCP				1.0 ± 1.3
SADCP/LADCP				0.7 ± 1.1
Altimetry			− 0.1 ± 0.2	− 0.1 ± 0.3

^EMF^Trends from cable transports corrected for the Earth Magnetic Field.

The trends (Sv per decade) are estimated using measurements from different platforms for different time intervals.

**Fig. 4:**
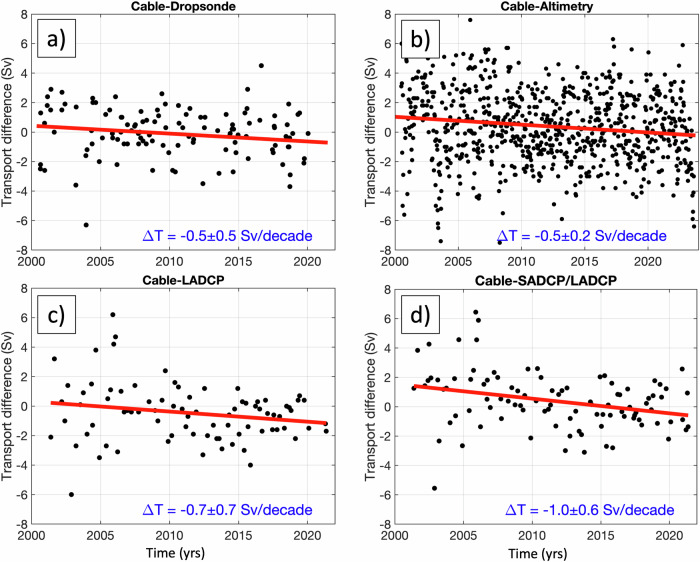
Differences between the concurrent estimates of the Florida Current volume transport. The differences are computed between the transports estimated from cable measurements (prior to corrections applied in this study) and from (**a**) dropsonde sections, (**b**) satellite altimetry measurements, (**c**) Lowered Acoustic Doppler Current Profiler (LADCP) sections, and (**d**) the combination of Shipboard and Lowered Acoustic Doppler Current Profilers (SADCP/LADCP) sections. The linear trends of the differences are shown by the red lines.

The obtained trends contrast with the overall trend and especially with the 2000–2023 trend determined from the cable data (Fig. 2; Table 1). To investigate the observed discrepancies between the trends in transports, we compute the concurrent differences between the cable transports and the transports estimated from individual ship sections (dropsonde, LADCP, and LADCP/SADCP) and from satellite altimetry, as well as their trends from 2000 to 2023 (Fig. 4). Ideally, if the cable data is well calibrated, we should expect the differences to be spread around the zero line with no or very small trends over the period of observations. Instead, all the differences exhibit negative trends, statistically significant for altimetry and LADCP/SADCP, indicating that the cable transports are consistently becoming lower than the transports inferred from all other observing platforms. This analysis indicates that there is likely an issue with the cable calibration, reasonably assuming that the ship section data yields more reliable transport estimates^8^. The fact that the cable transports are diverging from both the ship-based and altimetry-based transports in 2000–2023 raises a question of whether the statistically significant decline in the FC transport estimated from cable measurements in 2000–2023 (−0.7 ± 0.4 Sv per decade) is a real oceanic signal.

### Long-term change in voltages measured on the submarine cable

**Fig. 5:**
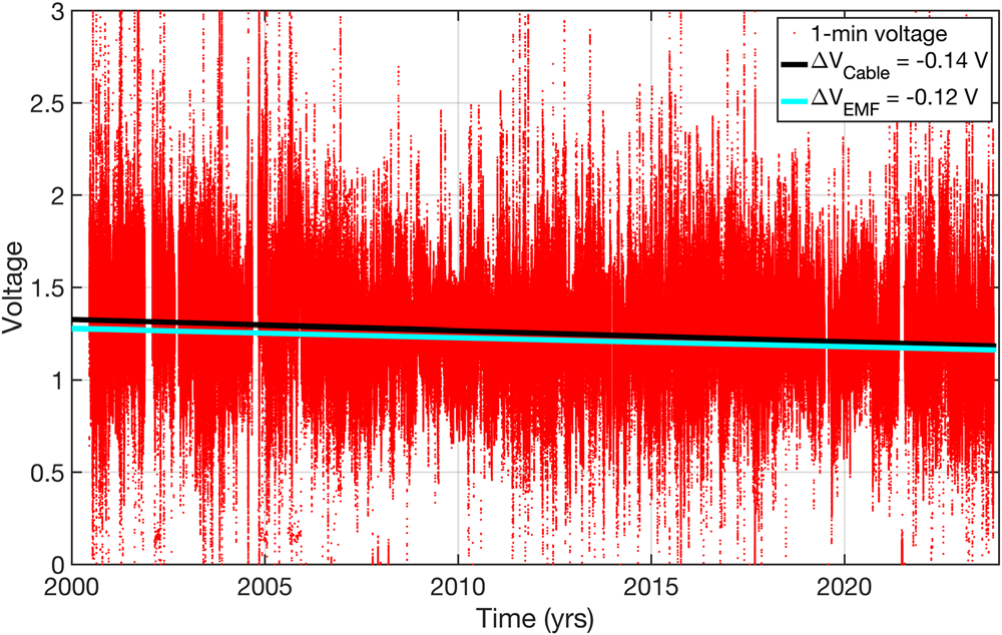
One-minute voltage recorded on the submarine cable between Florida and Grand Bahama Island. The linear change in the cable voltage from 2000 to 2023 is ΔV_Cable_ = −0.14 V (black line). The almost linear change in voltage due to the secular change in the vertical component of the Earth’s magnetic field (EMF) is ΔV_EMF_ = − 0.12 V (cyan line).

To investigate whether the decline in the FC transport observed in the cable data since 2000 is a real oceanic signal or a spurious drift due to data processing issues, the one-minute voltages measured on the cable in 2000–2023 were analyzed (Fig. 5). The voltages depend on the depth-averaged velocity, the vertical component of the EMF, the width of the strait at the cable location, the electrical conductivity of the ocean, and the conductance of sediments and upper conducting crust beneath the ocean (Eq. (1) in “Methods”; 7). The time-mean voltage in 2000–2023 is about 1.25 V. The voltage record exhibits a decreasing trend, with an overall 2000–2023 voltage reduction of about 0.14 V (black line Fig. 5).

Since the conductivity weighting (C_w_) and the conductance depth (H_e_) in Eq. (1) (“Methods”) can be considered constant, then the 2000–2023 decrease in voltage is either due to a decreasing FC transport (T_FC_) or the vertical component of the EMF (F_z_). The EMF is created by the planet’s moving, molten iron core. Therefore, its poles are not stationary but slowly and continuously move over geological time scales. Based on the International Geomagnetic Reference Field (IGRF) model^46^, the north magnetic pole has moved from the Canadian Archipelago towards Russia over the last century, and it is now located about 450 km away from the geographic North Pole. While in the early years of the cable record it was reasonable to assume that F_z_ was steady^4^, the 1982–1998 cable record was corrected for the secular change in the EMF by multiplying the motion-induced voltages by a factor inversely proportional to the secular change in F_z_^7^. However, for the reasons mentioned in Methods, the 2000–2023 cable record has not been corrected for the secular change in F_z_.

Using the IGRF model in the NOAA National Center for Environmental Information’s Magnetic Field Calculator (www.ngdc.noaa.gov/geomag/calculators/magcalc.shtml), we find that F_z_ in the Florida Straits (at 27°N, 79.5°W) decreased almost linearly from 3.96 × 10^−5^ Tesla (T) in 1999–3.60 × 10^−5^ T in 2023, equating to the total reduction of about 3.6 × 10^−6^ T (~150 nT per year) or about 9% from the initial value. The decreasing F_z_ in the Florida Straits leads to decreases in voltages and the amplitude of voltage variations measured by the cable (Eq. (1) in “Methods”). Assuming that the FC volume transport is constant (T_FC_ = 32 Sv), the conductivity weighting is 1.06, and the conductance depth is 1050 m^4^, the reduction in F_z_ would correspond to a voltage reduction of about 0.12 V measured on the cable (cyan line in Fig. 5). Thus, it appears that the voltage reduction due to the secular change in the EMF almost fully accounts for the actual voltage decrease measured on the cable in 2000–2023 (0.14 V). Given the calibration factor α = 24.42 Sv V^-1^, the voltage reduction of 0.12 V translates to a decrease of ~2.9 Sv in transport. This is a very large number that cannot be neglected, in particular when attributing a relatively small and marginally significant trend in the time series to a real climate signal. Recall that the 2000–2023 trend in the cable record was −0.7 ± 0.4 Sv per decade (Fig. 2, Table 1), which corresponds to a decline of about 1.7 Sv during this time.

### Corrected estimates of the Florida Current volume transport

From the analysis presented above, it is clear that with the increased length of the cable record it has become absolutely necessary to correct the 2000–2023 voltage and/or transport data for the secular change in the EMF. The easiest way to accomplish this would be to apply this correction offline to the estimated transports. However, the adjustments of the offsets β (Eq. (2) in “Methods”), performed by comparing the cable and ship section data during the times of known problems with the recording system, are likely to have partially compensated for the EMF change. Therefore, we modified the equation used to derive the transports from voltages to explicitly account for the secular change in F_z_ (Eq. (4) in “Methods”). We then recalibrated the obtained cable record and readjusted the offsets in problematic time intervals, before finally calculating the new transport estimates (see “Methods” for further details).

**Fig. 6:**
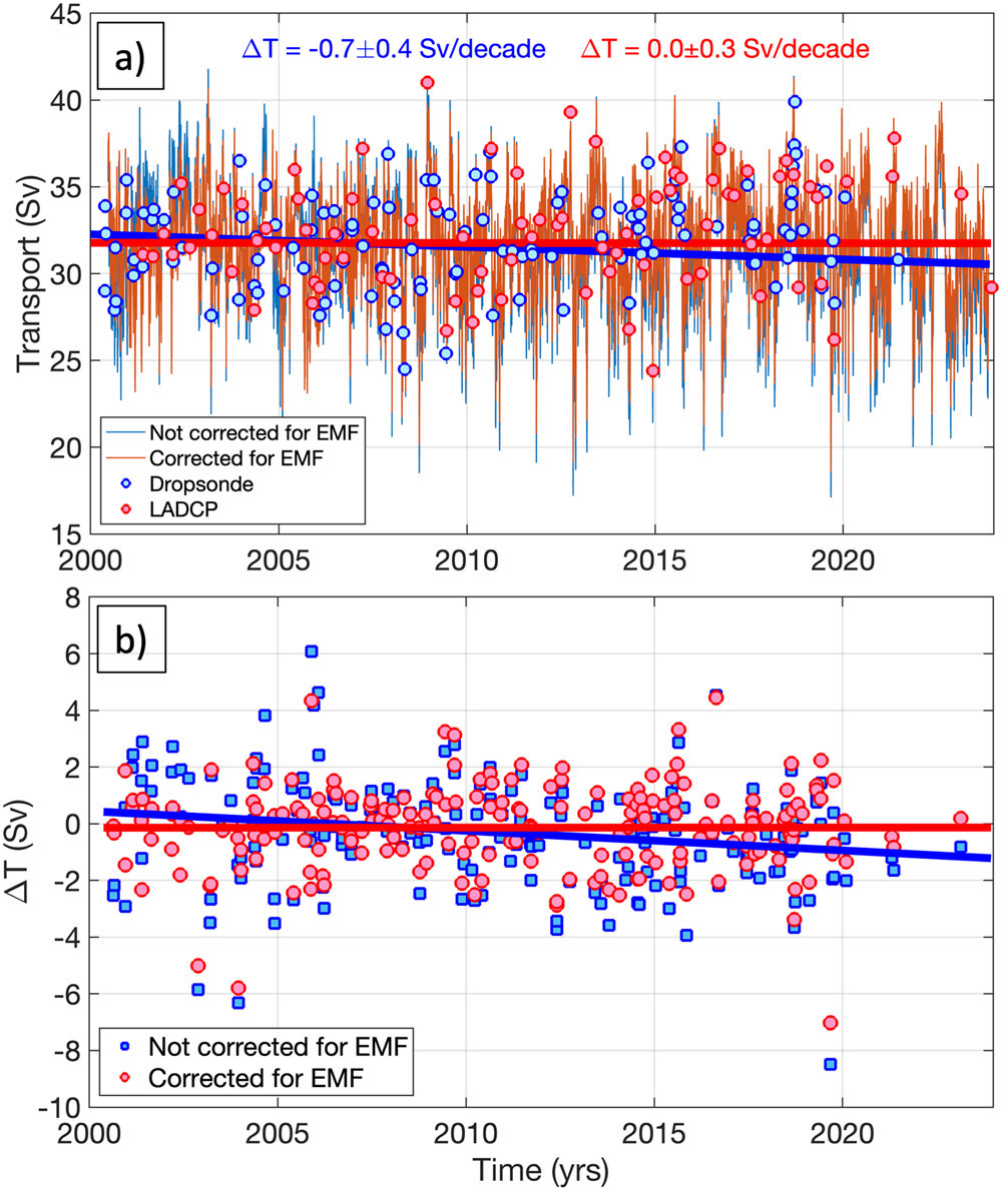
Florida Current (FC) volume transports corrected for the secular change in the Earth’s Magnetic Field (EMF). **a** The time series of the daily FC volume transport: (blue) not corrected for the secular change in the EMF, (red) corrected for the secular change in the EMF. The linear trends of the FC transport not corrected and corrected for the EMF are shown by the blue and red lines, respectively. **b** The differences between the cable and ship section transport for the cable data (blue squares) not corrected for the EMF and (red circles) corrected for the EMF. The linear trends of the differences (Δ*T*) not corrected and corrected for the EMF are shown by the blue and red lines, respectively.

Displayed in Fig. 6a are the obtained FC volume transports in 2000–2023 corrected for the secular change in the EMF. The 2000–2023 mean FC transport is 31.8 Sv and the standard deviation is 3.0 Sv. While there are some differences with the earlier record due to the recalibration of the offsets, in particular in 2000–2005, the most significant impact of applying the EMF correction is the elimination of the negative trend in the FC transports. The trend in 2000–2023 changed from −0.7 ± 0.4 Sv per decade in the earlier record to 0.0 ± 0.3 Sv per decade in the new record. When we combined the 2000–2023 cable record corrected for the EMF with the prior 1982–1998 record, we obtained the overall 1982–2023 trend of −0.1 ± 0.2 Sv per decade, i.e., statistically insignificant and four times smaller than for the cable record not corrected for the EMF ( − 0.4 ± 0.2 Sv per decade; Table 1). It is interesting to observe that the 1993–2023 trend in the cable record corrected for the EMF (−0.1 ± 0.3 Sv per decade) is the same with the 1993–2023 trend in the altimetry-derived FC transports ( − 0.1 ± 0.2 Sv per decade; Fig. 4b), and the 2000–2023 trends in the cable and altimetry transports are close within the uncertainty limits (Table 1).

To further assess the corrected and recalibrated cable transports, we again consider the differences between the concurrent cable and ship section transports (Fig. 6b). The time-mean and the RMS of the differences between the old (not corrected for the EMF) cable transports and the ship section transports are −0.3 Sv and 1.9 Sv, respectively. As has already been mentioned, the old cable transports in 2000–2023 were consistently becoming lower relative to the ship section transports. The differences between the old cable and ship section transports exhibit a negative trend of about −0.7 Sv per decade (blue line in Fig. 6b). In contrast, the difference between the new (corrected for the EMF) cable transports and the ship section transports has no trend (red line in Fig. 6b), meaning that the calibration of the corrected cable record has improved. Furthermore, the RMS difference between these estimates has reduced to 1.5 Sv. This analysis demonstrates that the newly corrected cable record is more accurate than the old record. Most importantly, by accounting for the secular change in the EMF, we show that the FC volume transport has not declined significantly over the past 40 years of observations, but in fact has remained remarkably stable.

### Implications for the Atlantic Meridional overturning circulation

The submarine cable has been a key component of the RAPID AMOC observing array at ~26.5°N since April 2004. Because applying a correction for the secular change in the EMF makes a significant change in the overall trend of the FC transport, the next logical question to address is how the newly corrected FC transport time series affects the AMOC calculation. The AMOC at ~26.5°N is composed of (i) the northward FC transport inferred from cable measurements, (ii) the predominantly northward near-surface meridional Ekman transport, and (iii) the southward upper-midocean transport (between the Bahamas and Africa) down to the deepest northward velocity at ~1100 m. Here, we recalculate the RAPID AMOC time series covering the period from April 2004 to February 2022 using the corrected FC transports and compare them to the previously published RAPID AMOC time series^47^.

**Fig. 7:**
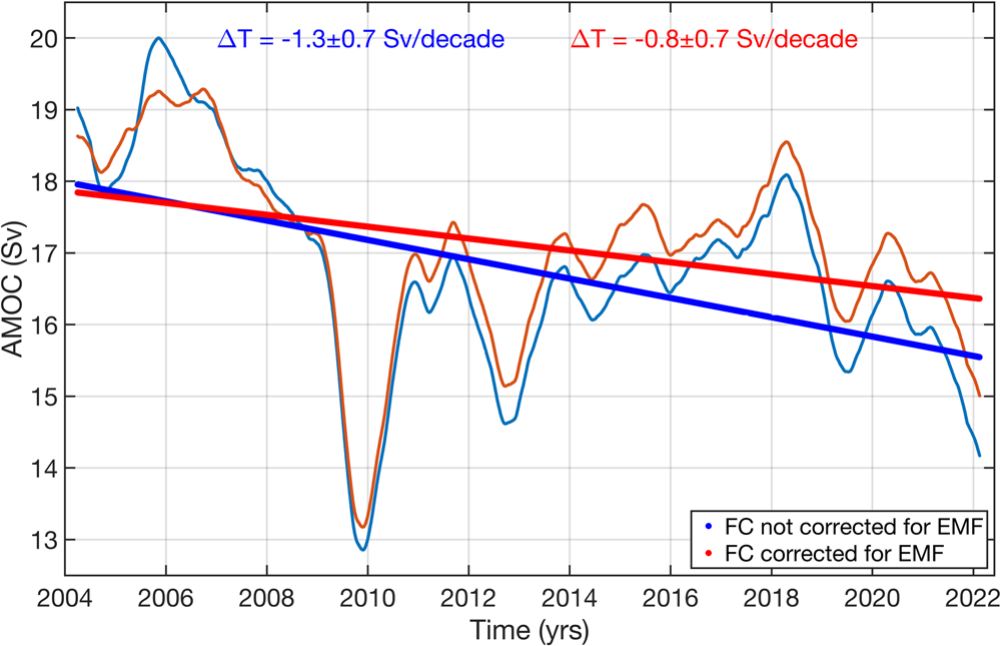
The northward transport of the Atlantic Meridional overturning circulation (AMOC), estimated from measurements collected at the RAPID observing array a ~26.5°N. The seasonal cycle was removed and the residual time series were low-pass filtered using a 2-year cutoff window. The blue curve and blue bold line (red curve and red bold line) show the AMOC time series (linear trend) computed with the Florida Current transport not corrected (corrected) for the secular change in the Earth Magnetic Field (EMF).

In the latter time series, the upper limb of the AMOC at ~26.5°N had a mean northward transport of about 16.8 Sv, composed of the mean FC transport of 31.3 Sv, the Ekman transport of 3.7 Sv, and the upper-midocean transport of −18.2 Sv. Observations at the RAPID array showcased that the AMOC was in its strongest state in the first years of observations (2004–2007; blue curve in Fig. 7). Then, the AMOC transport decreased abruptly reaching its record low value in 2009–2010. The recovery of the AMOC from the 2009–2010 dip was not complete and a state of reduced overturning persisted until 2017^21^. The negative AMOC tendency between 2004 and 2018 has been widely used in support of a likely AMOC slowdown, suggested by climate models and reconstructions based on ocean proxy data (e.g.^13,14,16,17^,). The overall 2004–2022 trend in the previously published RAPID time series is −1.3 ± 0.7 Sv per decade (blue curve in Fig. 7), thus also supporting the AMOC slowdown.

After the EMF correction is applied, the mean FC transport over the RAPID time interval increases to 31.7 Sv, and the mean AMOC transport increases to 17.1 Sv. The overall AMOC trend becomes −0.8 ± 0.7 Sv per decade, i.e., it is reduced by about 40% and becomes only marginally significant (red curve in Fig. 7). With the corrected FC transport, the AMOC is slightly reduced during the first years of the record, but it is increased starting from about 2009, becoming larger by almost 1 Sv compared to the earlier estimate toward the end of the time series. It is interesting to note that, using the revised FC transports, the second maximum in the AMOC time series observed in 2018 (~18.6 Sv) becomes much closer to high transports in 2005–2006 (~19.3 Sv). These findings provide a stronger support for the previously made conclusion that the observed AMOC time series at ~26.5°N showcases an interdecadal variability rather than a long-term decline.

## Discussion

There is growing scientific interest in how large-scale ocean circulation is changing or will change in the future in response to anthropogenic forcing. Particular attention is paid to the potential decline of the AMOC, fueled by the results based on state-of-the-art climate models and proxy data reconstructions. Although solid observational evidence is still lacking as direct observations of the AMOC are very short compared to climate time scales, some studies have used relatively small and often marginally significant trends in observational data as support for the notion of AMOC slowdown or even collapse (e.g.^14,15,17^,). Some conclusions based on observational products are oftentimes made without detailed consideration of how the raw data were processed. Arguments about the robustness of tendencies in observational data products usually rely only on statistical significance^39–41^. Yet, it is important to realize that an inherent nature of observational records is that they may be subject to spurious drifts and biases, caused by instrumentation and data processing issues. As a result, routine calibration, re-evaluation, and correction of observational data records, sometimes years after the initial data were released, is an integral and inevitable part of any long-term observational program. Therefore, small and marginally significant (in statistical sense) tendencies in data products should be treated with caution in any analysis of long-term changes.

In this study, we reassessed the 40-year record of the FC volume transport inferred from motion-induced voltages measured on submarine telecommunication cables in the Florida Straits since 1982. The cable record constitutes the longest observational record of a boundary current in existence. Therefore, these cable observations together with regular cable calibration cruises that directly measure the FC volume transport and water mass properties provide a unique and invaluable dataset to study climate variability in the North Atlantic on decadal time scales. Because the FC is the major contributor to the northward transport of both the upper-limb of the AMOC and the subtropical gyre circulation in the North Atlantic, it is reasonable to expect that on sufficiently long time scales a FC decline could indicate a slowdown in the AMOC and/or the gyre circulation. It should also be recalled that not all the western boundary flow is contained in the FC. A small, but highly variable, part of this flow is carried by the Antilles Current^48^.

The long history of cable measurements in the Florida Straits involved contributions from several generations of scientists. This has resulted in some differences in the processing methods of raw voltage data during the 1982–1998 and 2000–2023 periods (“Methods”). In addition, the use of different recording systems over time and recurrent instrument failures made it necessary to correct the cable voltages and inferred transports for spurious offsets using direct measurements of the FC transport carried out by ship surveys. After investigating the differences in data processing during the two cable epochs (1982–1998 and 2000–2023), we focused our attention on the impact of secular changes in the geomagnetic field on motion-induced voltages measured by the cable, which the earlier part of the record was explicitly corrected for. While we could not re-examine the 1982–1998 raw voltage record as part of it had been lost^3^, the small negative trend in the FC transport record is associated only with the 2000–2023 portion of the time series. The 1982–1998 transports have no meaningful trend. Since 2000, high-frequency geomagnetic signals have been effectively removed by applying a 3-day low-pass filter to the cable record. However, during this second epoch, the direct correction for the secular change in the geomagnetic field had previously not been done. In our analysis, we find that the observed decrease in voltages over the 2000–2023 period is almost fully accounted for by the secular change in the local EMF. Therefore, as the length of the cable record continues to grow, correcting it for secular EMF changes is crucial.

The 2000–2023 cable record was reprocessed by first correcting the cable voltages for the secular change in the EMF and then recalibrating the FC transports using ship section data. The corrected FC transport time series is made freely available through the WBTS project’s web page (https://www.aoml.noaa.gov/phod/wbts/). The most important outcome of this study is that the overall trend of the FC volume transport since 1982 has been revised. The updated trend estimate for the 1982–2023 period is −0.1 ± 0.2 Sv per decade, which is not statistically different from zero and 3–4 times smaller than it was thought previously before accounting for the EMF change. This result impacts the recently made assertion of the robust weakening of the FC^39^. Rather, it appears that the FC has remained remarkably stable over the 40 years of nearly continuous observations. Furthermore, the change in the FC cable transports necessitates a revision in the AMOC transports at ~26.5°N since 2004 derived from the RAPID array. The use of the corrected FC transport time series reduces the negative trend in the AMOC from 2004 to 2022 by about 40% and makes it only marginally significant in statistical sense.

The results of this study indicate that, if climate models are correct that the AMOC is slowing or will soon slow down, this slowdown has not yet been reflected in the FC and the AMOC transports, or the observational records are still too short to detect it with confidence. This is in accord with other observation-based studies^23–28^. Nevertheless, the likelihood of a future AMOC slowdown and the importance of both the FC and the AMOC in the regional and global climate variability emphasizes the value of sustained observations in the Florida Straits and in the subtropical North Atlantic at ~26.5°N. The existing records are just starting to resolve decadal-scale signals relevant to climate variability. Continued observations are thus necessary for detection and mechanistic understanding of climate-related changes and for validating and improving ocean and climate models.

## Methods

To analyze the long-term change in the Florida Current (FC) strength, we use the ~40-year (1982–2023) records of (i) the daily estimates of the FC volume transport inferred from voltages measured on submarine telecommunication cables between Florida and Grand Bahama Island and (ii) the snapshot transport estimates based on direct in situ measurements at the 27°N section. In addition, we use (iii) the transport estimates inferred from cross-stream sea surface height (SSH) differences measured by satellite altimetry in 1992–2023. All data are publicly available from the NOAA WBTS project website (https://www.aoml.noaa.gov/phod/wbts/).

### Submarine cable measurements

While a robust relationship between cross-stream transports and voltages and its oceanographic application are not guaranteed everywhere in the ocean, submarine cables installed between Florida and Grand Bahama Island (Fig. 1) have successfully been used to estimate the full-water-column transports across the cable^2–4,7^. The sustained, nearly continuous measurements started in March 1982 using the cable between Jupiter (Florida) and Settlement Point (Grand Bahama Island). Since 1993, a nearby cable between West Palm Beach (Florida) and Eight Mile Rock (Grand Bahama Island) has been used.

The estimates of the FC volume transport from voltage measurements on submarine cables are based on principles of electromagnetic induction. When ions present in seawater move with an ocean current through the Earth’s magnetic field (EMF), an electric field is induced perpendicular to the direction of water motion^49,50^. Because seawater is a conductive media, the induced electric field is proportional to the vertically averaged horizontal flow.

The local motion-induced, cross-stream voltages measured on a submarine cable can be approximated as follows^4^:

1 $$\Delta \phi \, \approx \, {C}_{w}\frac{{F}_{z}{T}_{{FC}}}{\left\langle {H}_{e}\right\rangle }$$

where ${C}_{w}=\left\langle \frac{\overline{\sigma v}}{\bar{\sigma }\bar{v}}\right\rangle$ is the conductivity weighting, σ is the electrical conductivity, v is the downstream velocity, <> represents a cross-channel average, and the overbar indicates depth-averaging, *F*_*z*_ is the vertical component of the EMF, ${T}_{{FC}}\left(t\right)={\int }_{0}^{{{\rm{W}}}}H\left(x\right)\bar{v}\left(x,\, t\right){dx}$ is the volume transport, *W* is the flow width, *t* is time, ${H}_{e}=H(1-\lambda )$ is the conductance depth, *H* is the water depth, and *λ* is the ratio of the solid earth’s conductance to the ocean’s conductance. If there were no electric currents flowing into the sea floor, then $\lambda$ would be zero. Based on collected profiling velocity and temperature data and assuming that conductivity is a linear function of temperature, the conductivity weighting *C*_*w*_ and the conductance depth *H*_*e*_ in Eq. (1) were estimated to be 1.06 ± 0.02 and 1050 ± 30 m, respectively, and thus assumed to be constant in time^4^. The Eq. (1) states that a cross-stream voltage depends on the depth-averaged velocity, the vertical component of the EMF, the stream width, the electrical conductivity of the ocean, and the conductance of the sediments above a resistive zone.

The motion-induced voltages measured on a submarine cable have been converted into volume transport estimates via linear transfer coefficients^7^:

2 $${T}_{{FC}}\left(t\right)=\alpha \Delta \phi \left(t\right)+\beta+\varepsilon (t)$$

where α = 24.42 Sv V^–1^ is the voltage calibration factor, *β* is the offset, and *ε* is the error. The calibration factor α and the offset *β* were originally determined by comparison with direct ship-based volume transport estimates obtained at the Jupiter – Settlement Point cable site in the 1980s^7,9^.

The present-day cable recording system consists of a computerized voltmeter that makes a measurement every minute and an automated, real-time processing system that involves a three-day lowpass filter with a 2nd order Butterworth filter passed both forward and back to remove tidal and high-frequency geomagnetic field variations^3^.

### Ship-borne measurements

The cable recording system is subject to hardware problems that result in data gaps and often in spurious drifts and offsets in the recorded voltages. Therefore, routine validation of cable-derived transports is required to ensure the stability of the recording equipment and the robustness of the cable record. This validation has been carried out with direct ship measurements at nine stations along the 27°N section (Fig. 1). In 1982–1988, the ship section data were collected by Pegasus profilers^5^, which were used in dropsonde mode after 1984 to cut the cost of maintaining acoustic sound sources. In the modern era, the direct measurements of the FC volume transport have been conducted with free-falling GPS dropsonde floats since 1991 (8-10 cruises per year) and with Lowered and Shipboard Acoustic Doppler Current Profilers (LADCP and SADCP) since 2001 (up to 6 cruises per year). As of June 2021, there were 248 dropsonde sections and 104 LADCP/SADCP sections. Unfortunately, in mid-2021 the Commonwealth of the Bahamas implemented new regulations for foreign marine scientific research clearance, which resulted in a cessation of research in Bahamian waters until December 2023.

The methods involved in calibrating and validating the cable records with dropsonde and LADCP measurements are well documented (e.g.^3,8^,). In this study, we also use the transport estimates based on the velocity sections produced by combining the LADCP and SADCP measurements. The SADCP data are processed and quality controlled using Common Ocean Data Access System (CODAS) developed by the University of Hawaii^51^. The main advantage of these combined sections is that they cover almost the entire strait from coast to coast, as opposed to the dropsonde and LADCP measurements that are collected at nine stations across the FC. Unlike the single LADCP velocity profiles associated with each of the nine sampling stations, the SADCP profiles incorporated into the LADCP/SADCP sections are averaged to 5-minute intervals along the cruise track. As the vessel’s speed and position varies during the hydrographic survey, so too does the spatial distribution and total number of SADCP profiles generated for each survey. Although it is reasonable to expect that the accuracy of LADCP/SADCP sections is greater than the accuracy of LADCP sections, we will conservatively assume that the accuracy of the LADCP/SADCP sections is equal to the accuracy of the LADCP sections. The accuracies of transport estimated from the Pegasus, dropsonde, and LADCP are presented in Table 2.

**Table 2:** Instrument accuracies

Time period	1982–1990	1991–1998	2000–2005		2006–present	
Instrument	Pegasus	Dropsonde	Dropsonde	LADCP	Dropsonde	LADCP
RMSE	1.4	3.2	2.2 (1.5)	2.9 (2.0)	1.7 (1.6)	1.6 (1.3)
Accuracy	1.0	1.3	0.8	1.3	0.8	1.3
Cable Error	1.0	2.9	2.1 (1.3)	2.6 (1.5)	1.5 (1.4)	0.9 (0.0)

Root mean squared differences (RMSE) between the cable- and ship-measured transports and individual accuracies of transports inferred from ship sections (8) and cable measurements. The cable error (bottom row) is inferred from RMSE and the instrument accuracy using Eq. (3) The values obtained from the comparison between the cable transports corrected for the Earth Magnetic Field and ship section transports are shown in brackets. The zero cable error in the 2006 to present time interval (in brackets) suggests that the accuracy of the estimates obtained from the Lowered Acoustic Doppler Current Profiler (LADCP) measurements is probably overestimated.

Using 104 Conductivity-Temperature-Depth (CTD) and LADCP/SADCP sections between 2001-2021, we recalculated the conductivity weighting parameter C_w_ in the Eq. (1) and obtained a time mean of 1.055 and a standard deviation of 0.014, i.e., almost identical to the one reported in ref. ^4^. There is a very small and statistically insignificant linear decrease of −0.003 over the 20 years of ship surveys and there is no correlation with the strength of the transport. This analysis demonstrates that the impact of ocean conductivity changes on voltages, for example during the FC warming since 2012^12,52,53^, is negligible.

### Cable data quality and accuracy

The cable record is nearly continuous except several relatively large gaps that occurred due to operational issues and instrument failures. The longest gap of 17-months occurred between October 1998 and June 2000 when there was a temporary lapse in the project funding and the building where voltage recording equipment was housed in West Palm Beach was closed down. A nearly 2-month gap occurred in September-October 2004, when Hurricanes Frances and Jeanne damaged the building housing similar equipment at Eight Miles Rock on Grand Bahama Island. More recently, there was a 1-month gap in July 2019 due to a lightning strike at the same facility in the Bahamas, and an almost 2-month gap in June-July 2021 due to an instrument failure. Shorter gaps are also present throughout the cable record. Overall, gaps constitute about 7% of the entire time series. Beginning in November 2023, a mechanical issue with the cable has resulted in an interruption of the daily FC transport time series. As of July 2024, the cable record has not yet been resumed, but we are working to resolve the problem as soon as possible.

**Fig. 8:**
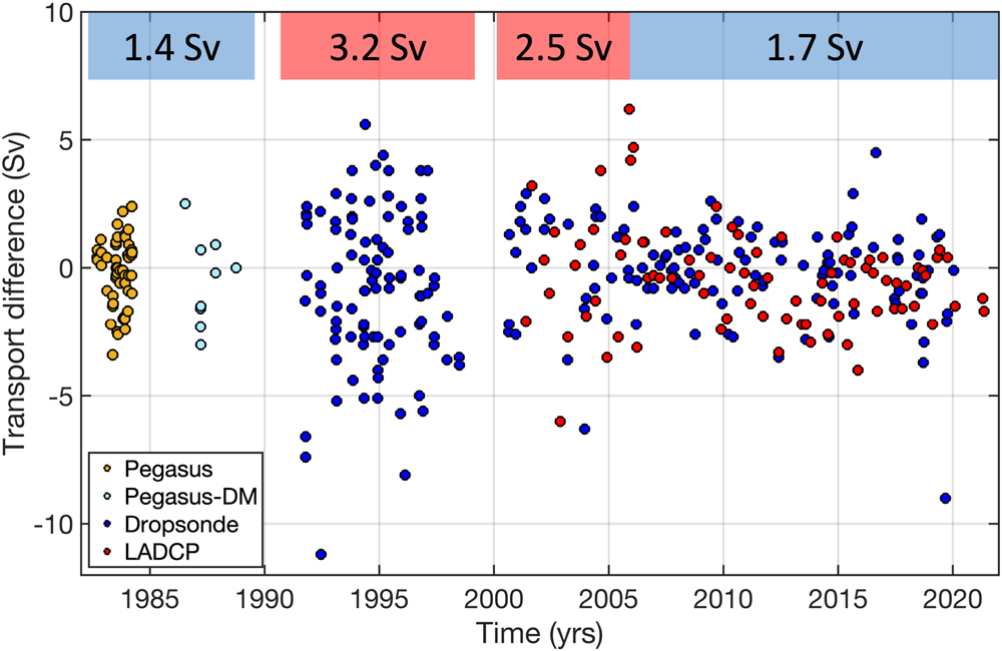
The differences between the concurrent transports estimated from the submarine cable (prior to corrections applied in this study) and ship section measurements. The colored circles show the following differences: (orange circles) cable—Pegasus, (light blue circles) cable—Pegasus in dropsonde mode (Pegasus-DM), (blue circles) cable—dropsonde, and (red circles) cable—Lowered Acoustic Doppler Current Profiler (LADCP). The root mean squared differences, computed for each of the four time intervals (1982–1988, 1991–1998, 2000–2005, 2006–2023), are shown at the top of the plot. The red rectangles delineate time intervals with problematic cable data quality, while the blue rectangles delineate time intervals with better cable data quality.

The accuracy of the cable-inferred transports, ${{{\rm{\varepsilon }}}}_{{{\rm{cable}}}}$, can be estimated by comparing them to the transports calculated from ship sections, using the published accuracies of the dropsonde and LADCP sections^8^:

3 $${\varepsilon }_{{cable}}={\left({\varepsilon }_{{total}}^{2}-{\varepsilon }_{{ships}}^{2}\right)}^{1/2}$$

where ${\varepsilon }_{{total}}$ is the root mean squared (RMS) difference between the transports obtained from cable measurements and from ship sections, and ${\varepsilon }_{{ships}}$ is the accuracy of transports estimated from ship section data. In Eq. (3) we assume that the two transport estimates are independent, which is true except for the relatively short period when ship section transports were used to determine the linear relationship between voltage and transport. The difference between the cable- and ship-based transports as well as the RMS differences over four time intervals: 1982–1988, 1991–1998, 2000–2005, and 2006–2023, prior to corrections applied in this study, are displayed in Fig. 8. We divide the record in this manner to isolate two periods characterized by the largest RMS differences and errors in the cable transports (shown in red in Fig. 8). These periods resulted from (i) the active use of the cable for telecommunication services in the 1990s^54^, and (ii) a problematic recording system in 2000–2005^3^.

The lowest RMS difference of ~1.4 Sv is observed in 1982–1988 using the Pegasus sections (Table 2). The errors of transports estimated from the Pegasus sections are of the order of 1 Sv^4,5^. This means that the error of the cable transports in the 1980s is also ~1 Sv. Using the observed RMS differences and published accuracies of the dropsonde sections^8^, the errors of the cable transports are 2.9 Sv in the 1990s, 2.1 Sv in 2000–2005, and 1.5 Sv in 2006–2021. A comparison with the LADCP sections since 2000 yields the errors of 2.6 Sv in 2000–2005 and 0.9 Sv in 2006–2021 (Table 2). The comparison between the cable and ship sections demonstrates that the quality of the cable record is not homogeneous over time, which needs to be taken into account when studying long-term changes of the FC.

### Differences in the cable data processing

It should be noted that cable voltages recorded before and after the 1998–2000 gap were processed somewhat differently. Dr. Jimmy Larsen, who led the Florida Current project from 1982 to 1998, applied corrections for high-frequency voltage variations induced in the ocean by the time varying ionospheric and magnetospheric electric currents (geomagnetic-induced voltages) and for the secular change in the EMF^7^. These corrections were derived from the shore-based magnetic data at San Juan (Puerto Rico) and Fredericksburg (Virginia) observatories. Because these observatories are located far from the Straits of Florida, when cable voltage measurements resumed in 2000 by the WBTS program, it was decided that smoothing the data with a 3-day low-pass filter would be sufficient to remove the high-frequency geomagnetic-induced voltages and the impact of tides. Additionally, as the WBTS project began collecting voltage data, the initial record was relatively short, and it was reasonably assumed that geomagnetic-induced secular changes were small. Thus, since the 2000, no correction for the secular change in the EMF has been applied.

However, regular calibration cruises have continued to be used to detect systematic biases between the cable and ship section transports. These biases occurred during the time intervals usually following the replacements and failures of the recording system that happened several times, mostly in 2000–2005 (e.g., when it was destroyed by hurricanes in 2004). Once detected, these biases are minimized by calculating the mean difference between the concurrent ship section and cable transports and adding it to the prior value of *β* in Eq. (2) This adjustment to *β* represents a piecewise constant function, different from zero only in problematic time intervals. The calibration factor α has remained unchanged through the entire 1982–2023 record. Despite the different processing methods used during the different cable periods, they did not result in particularly different spectra^3^.

It has also been assumed that the adjustments of the offset *β* in problematic time intervals could effectively remove the impact of the secular change in the EMF. Nevertheless, a problem with this latter approach is that relatively small secular changes in the geomagnetic-induced voltages can only be detected when the cable record is sufficiently long. This is one of the reasons why it took about 23 years to realize that an explicit correction for the EMF is necessary to avoid misinterpretation of the trend. Therefore, in this study, we first correct the entire modern era cable voltage data (since 2000) for the secular change in the EMF, then recalibrate cable transports using recent ship section data, and finally readjust *β* in problematic time intervals.

### Correcting voltage data for the secular change in the EMF

The yearly vertical component of the EMF, *F*_z_, used in this study comes from the latest, thirteenth generation International Geomagnetic Reference Field (IGRF). The IGRF is a set of spherical harmonic coefficients, derived from observations recorded by satellites, ground observatories, and magnetic surveys^46^. These coefficients are used as input into a mathematical model, which describes the large-scale, time-varying EMF. Because of the secular variation in the EMF, the IGRF models are specified every 5 years (epochs), with linear interpolation used for intermediate times.

Errors in the coefficients lead to uncertainties in the resulting model field, which are summarized in terms of the corresponding RMS vector uncertainty of the field when averaged over the Earth’s surface^55^. It is suggested that after 2000, when satellite data is available, the RMS vector uncertainty is close to 10 nT. It should be noted that at any specific location, a 10 nT vector error is shared between the three orthogonal components (*x*, *y*, and *z*). For example, a 10 nT global RMS vector error can correspond to global RMS values of about 5, 5, and 7 nT for *F*_*x*_, *F*_*y*_, and *F*_*z*_, respectively^56^. Since 2000, the local *F*_*z*_ in the Florida Straits (at 27°N, 79.5°W) has been declining at a rate of ~150 nT per year. Hence, the RMS error constitutes about 5% of the typical yearly decline and can be neglected.

According to Eq. (1) the secular change in the EMF has caused a negative trend in recorded voltages and a decrease in the magnitude of voltage variations. In the 1982–1998 record, these effects were removed by multiplying the motion-induced voltages by a factor inversely proportional to *F*_*z*_^7^. Following the same methodology in this study, we computed the FC volume transports since 2000 using a modified version of Eq. (2)

4 $${T}_{{FC}}\left(t\right)=\alpha \frac{{F}_{z}^{0}}{{F}_{z}\left(t\right)}\varDelta \phi (t)+\beta+\varepsilon (t)$$

in which ${F}_{z}^{0}$ is the reference vertical component of the EMF. ${F}_{z}^{0}$ was set to *F*_*z*_ on Jan 1, 2019, which is about the temporal mid-point of ship surveys used to recalibrate cable voltages into volume transports.

### Recalibration of cable transports and their accuracy

To calculate transports using Eq. (4) we recalibrated linear transfer coefficients, *α* and *β*, using modern era voltage measurements and ship section data. Recall that the original *α* was estimated for the old cable between Jupiter and Settlement Point, so the recalibration for the modern cable between West Palm Beach and Eight Miles Rock is well justified. For the recalibration, we chose a subset of cable voltages and ship section transports between January 1, 2016 and November 12, 2023. In total, there were 44 ship sections conducted during this time interval, consisting of 22 dropsonde and 22 LADCP sections. The newly calculated linear transfer coefficients are: *α* = 22.5 Sv V^-1^ and *β* = 4.2 Sv. These coefficients will be used for future cable measurements until we determine that another calibration and/or offset adjustment is required, based on comparisons between the cable and ship section transports.

The implementation of the EMF correction and the recalibration of cable transports has led to a reduction in cable error estimates since 2000 (Table 2). The RMS difference between the concurrent cable and ship section transports in 2000–2023 has been decreased from 1.9 Sv to 1.5 Sv. In 2000–2005, the RMS difference between the cable and the dropsonde (LADCP) section transports has been decreased from 2.2 Sv (2.9 Sv) to 1.5 Sv (2.0 Sv), resulting in a conservative cable error of 1.5 Sv. Since 2006, the cable error has become 1.4 Sv based on the comparison with dropsonde sections. The RMS of the difference between the cable and LADCP section transports in 2006–2023 has decreased to 1.3 Sv, so that it has become equal to the published accuracy of LADCP section transports. This indicates that the accuracy of LADCP section transports is probably overestimated and needs to be revisited in a future study.

### Transports inferred from SSH gradients

While the cable recording system provides cost-effective means for determining the FC volume transport in near real-time, there have been efforts to explore the utility of alternative (backup) observing platforms for inevitable system failures or possible future cable breaks^44,57^. As part of the WBTS project, a pair of bottom pressure gauges have been deployed in shallow waters (~12 m depth) on both sides of the Straits of Florida at 27°N since July 2008 (yellow stars in Fig. 1). Cross-stream pressure differences calibrated into the FC transports can explain up to 55% of the cable-derived FC transport variance^57^. However, because the pressure gauges are subject to instrument drifts, data gaps, and their records are relatively short, they are still of limited use in determining long-term changes in the FC volume transport. On the other hand, satellite altimetry has provided gap-free and homogeneous-quality along-track sea surface height profiles across the FC roughly every 10 days since November 1992 (the satellite track used for the FC transport estimates is shown in Fig. 1). Similar to the bottom pressure gauges, the FC transports inferred from the cross-stream SSH gradients measured by altimetry explain about 60% of the 10-day cable-derived FC transport variance^44^. The accuracy of transports estimated from satellite altimetry data is 2.1 Sv, which is based on the comparisons with both the cable and the ship sections. The main advantage of the remote sensing of the FC with satellite altimetry over in situ observations (cable, ship sections, and bottom pressure gauges) is that it is not impacted by adverse weather conditions.

### Statistical analysis

The long-term changes in the FC strength from the cable and other observing platforms were computed by least squares fit of the linear trend model. The uncertainty in the trend represents the formal error of the fit (deviations of observations from the trend), and the random measurement error using the 95% confidence interval. Recall that the random measurement errors for the cable and ship section measurements are time dependent. In this study, for estimating the uncertainties of trends in the cable data, we used the conservative measurement errors for the cable transports shown in Table 2, i.e., 1 Sv in the 1980s, 2.9 Sv in the 1990s, 2.6 Sv in 2000–2005, and 1.5 Sv since 2006. The contribution of random measurement errors to the trend uncertainty was estimated using a Monte Carlo style approach for each uncertainty estimate by generating 10,000 random time series, with variable standard deviations equal to the estimated measurement errors in different time intervals as specified in Table 2.

## Supplementary information

Peer Review File

## Data Availability

All the data used in this work is publicly available, and the references to this data are provided in the main text. The Florida Current volume transports estimated from the cable and ship section measurements are available from the Western Boundary Time Series project web page: https://www.aoml.noaa.gov/phod/floridacurrent/data_access.php. The Atlantic Meridional Overturning Circulation transport data are available from the RAPID project web page: https://rapid.ac.uk/data.php.

## Code availability

The codes written to analyze data for this work are available at https://codeocean.com/capsule/1291301/tree/v1.

## References

[1] Archer, M. R., Shay, L. K. & Johns, W. E. The surface velocity structure of the Florida Current in a jet coordinate frame. *J. Geophys. Res.: Oceans* **122**, 9189–9208 (2017).

[2] Baringer, M. O. & Larsen, J. C. Sixteen years of Florida Current transport at 27°N. *Geophys. Res. Lett.* **28**, 3179–3182 (2001).

[3] Meinen, C. S., Baringer, M. O. & Garcia, R. F. Florida current transport variability: An analysis of annual and longer-period signals. *Deep-Sea Res.* **57**, 835–846 (2010).

[4] Larsen, J. C. & Sanford, T. B. Florida current volume transports from voltage measurements. *Science* **227**, 302–304 (1985). 17742101 10.1126/science.227.4684.302

[5] Molinari, R. L., Wilson, W. D. & Leaman, K. Volume and heat transports of the Florida current: April 1982 through August 1983. *Science* **227**, 295–297 (1985). 17742099 10.1126/science.227.4684.295

[6] Leaman, K. D., Molinari, R. L. & Vertes, P. S. Structure and variability of the Florida current at 27N: April 1982–July 1984. *J. Phys. Oceanogr.* **17**, 565–583 (1987).

[7] Larsen, J. C. Transport and heat flux of the Florida current at 27N derived from cross-stream voltages and profiling data: theory and observations. *Philos. Trans. R. Soc. Lond. A* **338**, 169–236 (1992).

[8] Garcia, R. F. & Meinen, C. S. Accuracy of Florida current volume transport measurements at 27°N using multiple observational techniques. *J. Atmos. Ocean. Technol.* **31**, 1169–1180 (2014).

[9] Spain, P. F., Dorson, D. L. & Rossby, H. T. PEGASUS: A simple, acoustically tracked velocity profiler. *Deep-Sea Res.* **28A**, 1553–1567 (1981).

[10] Richardson, W. S. & Schmitz, W. J. A technique for the direct measurement of transport with application to the Straits of Florida. *J. Mar. Res.* **23**, 172–185 (1965).

[11] Frajka-Williams E., Ansorge I. J., Baehr J., Bryden H. L., and co-authors. Atlantic meridional overturning circulation: Observed transports and variability, Front. Mar. Sci., 10.3389/fmars.2019.00260 (2019).

[12] Johns, W. E. et al. Towards two decades of Atlantic Ocean mass and heat transports at 26.5N,. *Philos. Trans. R. Soc. A.* **381**, 20220188 (2023). 10.1098/rsta.2022.0188 PMC10590663 37866389

[13] Weijer, W., Cheng, W., Garuba, O. A., Hu, A. & Nadiga, B. T. CMIP6 models predict significant 21st century decline of the Atlantic meridional overturning circulation. *Geophys. Res. Lett.* **47**, e2019GL086075 (2020).

[14] Rahmstorf, S. et al. Exceptional twentieth-century slowdown in Atlantic Ocean overturning circulation. *Nat. Clim. Change* **5**, 475–480 (2015).

[15] Boers, N. Observation-based early-warning signals for a collapse of the Atlantic Meridional Overturning Circulation. *Nat. Clim. Chang.* **11**, 680–688 (2021).

[16] Caesar, L. et al. Observed fingerprint of a weakening Atlantic Ocean overturning circulation. *Nature* **556**, 191–196 (2018). 29643485 10.1038/s41586-018-0006-5

[17] Caesar, L., McCarthy, G. D., Thornalley, D. J. R., Cahill, N. & Rahmstorf, S. Current Atlantic Meridional Overturning Circulation weakest in last millennium. *Nat. Geosci.* **14**, 118–120 (2021).

[18] Kilbourne K. H. et al. Atlantic circulation change still uncertain. *Nat. Geosci.*, 10.1038/s41561-022-00896-4 (2022).

[19] Little, C. M., Zhao, M. & Buckley, M. W. Do surface temperature indices reflect centennial- timescale trends in Atlantic Meridional Overturning Circulation strength? *Geophys. Res. Lett.* **47**, e2020GL090888 (2020).

[20] He, C. et al. A North Atlantic warming hole without ocean circulation. *Geophys. Res. Lett.* **49**, e2022GL100420 (2022).

[21] Smeed, D. A. et al. The North Atlantic Ocean is in a state of reduced overturning. *Geophys. Res. Lett.* **45**, 1527–1533 (2018).

[22] Moat, B. I. et al. Pending recovery in the strength of the meridional overturning circulation at 26° N,. *Ocean Sci.* **16**, 863–874 (2020).

[23] Rossby, T., Palter, J. & Donohue, K. What can hydrography between the New England Slope, Bermuda and Africa tell us about the strength of the AMOC over the last 90 years? *Geophys. Res. Lett.* **49**, e2022GL099173 (2022).

[24] Rossby, T. et al. Oleander is more than a flower: Twenty-five years of oceanography aboard a merchant vessel. *Oceanography* **32**, 126–137 (2019).

[25] Rossby, T., Flagg, C. N., Donohue, K., Sanchez-Franks, A. & Lillibridge, J. On the long-term stability of Gulf Stream transport based on 20 years of direct measurements. *Geophys. Res. Lett.* **41**, 114–120 (2014).

[26] Caínzos, V. et al. Thirty years of GO-SHIP and WOCE data: Atlantic overturning of mass, heat, and freshwater transport. *Geophys. Res. Lett.* **49**, e2021GL096527 (2022).

[27] Fu, Y., Li, F., Karstensen, J. & Wang, C. A stable Atlantic meridional overturning circulation in a changing North Atlantic Ocean since the 1990s. *Sci. Adv.* **6**, eabc7836 (2020). 33246958 10.1126/sciadv.abc7836 PMC7695472

[28] Worthington, E. L. et al. A 30-year reconstruction of the Atlantic meridional overturning circulation shows no decline. *Ocean Sci.* **17**, 285–299 (2021).

[29] Smeed, D. A. et al. Observed decline of the Atlantic meridional overturning circulation 2004–2012. *Ocean Sci.* **10**, 29–38 (2014).

[30] Roberts, C. D., Jackson, L. & McNeall, D. Is the 2004-2012 reduction of the Atlantic meridional overturning circulation significant? *Geophys. Res. Lett.* **41**, 3204–3210 (2014).

[31] Chen, C., Wang, G., Xie, S.-P. & Liu, W. Why does global warming weaken the Gulf Stream but intensify the Kuroshio? *J. Clim.* **32**, 7437–7451 (2019).

[32] Dong, S., Baringer, M. O. & Goni, G. J. Slow Down of the Gulf Stream during 1993–2016. *Sci. Rep.* **9**, 6672 (2019). (2019). 31040298 10.1038/s41598-019-42820-8 PMC6491472

[33] Volkov, D. L. Meridional overturning circulation and heat transport in the Atlantic Ocean, [in State of the Climate in 2020]. *Bull. Am. Meteor. Soc.* **102**, S176–S179 (2021).

[34] Volkov, D. L. Atlantic meridional overturning circulation and associated heat transport, [In: State of the Climate 2019]. *Bull. Am. Met. Soc.* **101**, S159–S163 (2020).

[35] Baringer, M. O. Meridional overturning and oceanic heat transport circulation observations in the North Atlantic Ocean, [in State of the Climate in 2017]. *Bull. Am. Meteor. Soc.* **99**, S91–S94 (2018).

[36] Baringer, M. O. Meridional overturning and oceanic heat transport circulation observations in the North Atlantic Ocean, [in State of the Climate in 2016]. *Bull. Am. Meteor. Soc.* **98**, S84–S87 (2017).

[37] Baringer, M. O. Meridional overturning circulation observations in the North Atlantic Ocean, [In: State of the Climate in 2014]. *Bull. Am. Met. Soc.* **96**, S78–S80 (2016).

[38] Baringer, M. O. et al. Meridional heat transport in the Atlantic Ocean, [In: State of the Climate in 2013]. *Bull. Am. Met. Soc.* **95**, S69–S71 (2014).

[39] Piecuch, C. G. & Beal, L. M. Robust weakening of the Gulf Stream during the past four decades observed in the Florida Straits. *Geophys. Res. Lett.* **50**, e2023GL105170 (2023).

[40] Pietrafesa, L. J., Bao, S., Gayes, P. T., Carpenter, D. D. & Kowal, J. C. Variability and trends of the Florida current and implications for the future of the Gulf Stream. *J. Coast. Res.* **38**, 1096–1103 (2022).

[41] Park, J. & Sweet, W. W., Accelerated sea level rise and Florida Current transport. *Ocean Sci.* **11**, 607–615 (2015).

[42] Piecuch, C. G. Likely weakening of the Florida Current during the past century revealed by sea-level observations. *Nat. Commun.* **11**, 3973 (2020). 32770036 10.1038/s41467-020-17761-w PMC7415143

[43] Pujiana, K. et al. Genesis of the Gulf Stream subseasonal variability in the Florida Straits. *J. Geophys. Res.: Oceans* **128**, e2022JC018555 (2023).

[44] Volkov, D. L. et al. Inferring Florida current volume transport from satellite altimetry. *J. Geophys. Res.: Oceans* **125**, e2020JC016763 (2020).

[45] Volkov D. L. et al. Meridional overturning circulation and heat transport in the Atlantic Ocean, [in State of the Climate in 2022]. *Bull. Amer. Meteor. Soc*., **104**, S181-S184, 10.1175/BAMS-D-23-0076.2.

[46] Alken P. et al. International geomagnetic reference field: The thirteenth generation. Earth Planets Space 73, 10.1186/s40623-020-01288-x (2021).

[47] Moat B. I. et al. Atlantic meridional overturning circulation observed by the RAPID-MOCHA-WBTS (RAPID-Meridional Overturning Circulation and Heatflux Array-Western Boundary Time Series) array at 26N from 2004 to 2022 (v2022.1). *NERC EDS British Oceanographic Data Centre NOC*. 10.5285/04c79ece-3186-349a-e063-6c86abc0158c (2023).

[48] Meinen, C. S. et al. Structure and variability of the Antilles Current at 26.5°N. *J. Geophys. Res. Oceans* **124**, 3700–3723 (2019).

[49] Sanford, T. B. Motionally-induced electric and magnetic fields in the sea. *J. Geophys. Res.* **76**, 3476–3492 (1971).

[50] Stommel, H. The theory of the electric field induced in deep ocean currents. *J. Mar. Res.* **7**, 386–392 (1948).

[51] Hummon J. M. et al. CODAS+UHDAS Documentation (282:c289b3782d3a). *Zenodo*, 10.5281/zenodo.8371260 (2023).

[52] Domingues, R., Goni, G., Baringer, M. & Volkov, D. What caused the accelerated sea level changes along the US East Coast during 2010–2015? *Geophys. Res. Lett.* **45**, 13–367 (2018).

[53] Volkov, D. L., Lee, S.-K., Domingues, R., Zhang, H. & Goes, M. Interannual sea level variability along the southeastern seaboard of the United States in relation to the gyre‐scale heat divergence in the North Atlantic. *Geophys. Res. Lett.* **46**, 7481–7490 (2019).

[54] Larsen, J. C. Transport measurements from in-service undersea telephone cables. *IEEE J. Ocean. Eng.* **16**, 313–318 (1991).

[55] Lowes, F. J. An estimate of the errors of the IGRF/DGRF fields 1945–2000. *Earth Planet Sp.* **52**, 1207–1211 (2000).

[56] Lowes F. J. IGRF Health warning, errors, and limitations. IAGA Working Group VMOD, https://www.ncei.noaa.gov/products/international-geomagnetic-reference-field/health-warning (2022).

[57] Meinen C. S., Garcia R. F., & Smith R. H. Evaluating pressure gauges as a potential future replacement for electromagnetic cable observations of the Florida Current transport at 27°N. *J. Oper. Oceanogr.*, 1–11, 10.1080/1755876X.2020.1780757 (2020).

