# Author Correction: Florida Current transport observations reveal four decades of steady state

**DOI:** 10.1038/s41467-025-58976-z

**Published:** 2025-04-22

**Authors:** Denis L. Volkov, Ryan H. Smith, Rigoberto F. Garcia, David A. Smeed, Ben I. Moat, William E. Johns, Molly O. Baringer

**Affiliations:** 1 Cooperative Institute for Marine and Atmospheric Studies, University of Miami, Miami, FL USA; 2 NOAA Atlantic Oceanographic and Meteorological Laboratory, Miami, FL USA; 3 National Oceanography Centre, Southampton, UK; 4 Rosenstiel School of Marine, Atmospheric, and Earth Science, University of Miami, Miami, FL USA

**Keywords:** Physical oceanography, Physical oceanography

Correction to: *Nature Communications* 10.1038/s41467-024-51879-5, published online 05 September 2024

The copyright holder for this article was incorrectly given as ‘The Author(s)’ but should have been ‘National Oceanography Centre and University of Miami. Parts of this work were authored by US Federal Government authors and are not under copyright protection in the US; foreign copyright protection may apply’.

In addition, this article was originally published with the incorrect license ‘CC BY-NC-ND’; it should have been ‘CC BY’.

The original article has been corrected.

