# Supplementary material for: Florida Current transport observations reveal four decades of steady state
Source: Nat Commun. 2024 Sep 5;15:7780. doi: 10.1038/s41467-024-51879-5 (PMC11377814; doi:10.1038/s41467-024-51879-5)
Supplement: Supplementary file 1 — Peer Review File [file 41467_2024_51879_MOESM1_ESM.pdf]

nature portfolio

Peer Review File

Florida Current transport observations reveal four decades of steady state

Open Access This file is licensed under a Creative Commons Attribution 4.0 International License, which permits use, sharing, adaptation, distribution and reproduction in any medium or format, as long as you give appropriate credit to the original author(s) and the source, provide a link to the Creative Commons license, and indicate if changes were made. In the cases where the authors are anonymous, such as is the case for the reports of anonymous peer reviewers, author attribution should be to 'Anonymous Referee' followed by a clear attribution to the source work. The images or other third party material in this file are included in the article's Creative Commons license, unless indicated otherwise in a credit line to the material. If material is not included in the article's Creative Commons license and your intended use is not permitted by statutory regulation or exceeds the permitted use, you will need to obtain permission directly from the copyright holder. To view a copy of this license, visit http://creativecommons.org/licenses/by/4.0/.

REVIEWER COMMENTS

Reviewer #1 (Remarks to the Author):

The authors show that the observed post-2000 decline in Florida Current transport (FCT) by underwater cables is primarily driven by the secular change in the geomagnetic field. After correcting for the geomagnetic effects, FCT has remained stable without significant trends over the past ~40 years. Considering FCT is an important component of the Atlantic meridional overturning circulation (AMOC), it also indicates a weaker decline in AMOC. The Florida Current is critical for meridional heat and mass transportation in the climate system. This manuscript provides important observational evidence about its long-term trend since 1982, which is important for understanding the internal variability of ocean circulations and climate change. However, some necessary details about the geomagnetic correction and FC observations are missing. Overall, the manuscript is well-written and potentially interesting for a wide range of audiences. I recommend the manuscript be considered after a major revision. My specific comments are listed below:

Major concerns

1. Line 86-89: I believe records from calibration cruises were also adopted in estimating FCT before 1998. Thus, I am wondering why the geomagnetic correction is applied for the period 1982-1998 but not for 2000-present before the work presented in the manuscript?

2. The trend in the vertical component of the Earth's Magnetic Field (EMF) is fundamental to the geomagnetic correction and the conclusions of this manuscript. What is the uncertainty of the trend in the vertical component of EMF (F_z)? Is the uncertainty comparable with the trend itself? A detailed discussion on this matter is necessary to establish the reliability of the conclusions.

3. Line 287-289: The Florida Current is not only the surface limb of AMOC, but also the western boundary component of the wind-driven circulations in the subtropical gyre. A Florida Current decline may indicate either a weakened AMOC or a weakened subtropical gyre. Other factors, such as changes in local wind stress or warming coastal water, may also affect FCT. Therefore, a decline in FCT is not necessarily linked to an AMOC slowdown. I recommend the authors add some brief discussions about the source of FC and how to interpret trends in FCT.

4.Line 371: The old FCT was estimated from equation (2) without the geomagnetic correction. Differences between the cable observations and ship measurements, including the long-term trend introduced by changes in the geomagnetic field, should be compensated by β(t), as mentioned in lines 461-464. With the correction term β(t), I expect that the FCT trends derived from cables should be very similar to the trends from calibration cruises. Thus, why are changes in the geomagnetic field not corrected by β(t) and the calibration cruises as expected, and the additional geomagnetic correction required?

5. Several cables were used to monitor FCT. Are the observed voltages affected by the material or diameter of the cables? Are the FCT, except the geomagnetic correction, derived from the voltages by exactly the same method before and after 2000? How do the differences, if applicable, affect the FCT trend? Since the manuscript is about the long-term trend of FCT, the consistency and differences of how the FCT is derived from cable measurements during different periods are very important and should be documented with more details.

Minor concerns

1. Line 108-109: The authors mentioned that the decreasing trend of FCT is “largely due to the most recent 2-3 years”. I am curious about how much will the geomagnetic correction affect FCT in the most recent 2-3 years?

2.In the geomagnetic correction, is it a linear Fz trend adopted for in eq (4)?

3. The water conductivity is affected by its temperature. I am wondering whether the warming trend in Florida Current affected its transport derived from the cables?

4. Line 408: “that” -> “than”.

Reference

Chen, C., Wang, G., Xie, S.-P., Liu, W., 2019. Why Does Global Warming Weaken the Gulf Stream but Intensify the Kuroshio? J. Clim. 32, 7437–7451. https://doi.org/10.1175/JCLI-D-18-0895.1

Reviewer #1 (Remarks on code availability):

The codes are easy to understand, and I can use them to reproduce the Florida Current transport with geomagnetic correction. The authors also provide cruise observations. With those data, I can reproduce critical results presented in the manuscript.

On the other hand, the code attached is more like the data used in the manuscript with a detailed document. It provides the voltage records and the coefficients to convert voltages into transports, with little information about how those coefficients are created.

Overall, the code provides additional details about the work, which would be a useful reference for the community

Reviewer #2 (Remarks to the Author):

Summary

The Florida Current (FC) time series is a unique oceanographic dataset in terms of its length and temporal coverage. It is also uniquely important, as FC transport is the largest component of the Atlantic Meridional Overturning Circulation (AMOC) as measured by the RAPID array—itself the longest continuous measurement of the AMOC. The significance of both time series derives from the large impact the AMOC has on Northern Hemisphere climate. There are good physical reasons, as well as numerous climate model forecasts, that imply that the AMOC is likely to slow in response to climate change. A number of previous studies have argued that this slowdown is already observable. Often the most compelling evidence offered for a currently slowing AMOC is the RAPID time series itself, which shows a significantly downward trend of ~8% per decade. Much of the downward trend in the RAPID time series is ultimately due to a downward trend in the FC time series. This manuscript makes a compelling argument that this FC trend is spurious and that correcting this spurious trend renders the AMOC trend only marginally significant.

As conducting fluid flowing through the Earth's magnetic field, the FC produces an electric field that produces a voltage drop across the Straits of Florida that is measured by undersea cables running between Florida and The Bahamas. The voltage is proportional to the product of FC transport and the local geomagnetic field strength. The transport-voltage relation is calibrated using regular hydrographic surveys. It was assumed that this calibration would compensate for fluctuations in the local geomagnetic field, but the authors find that calibration is only able to compensate for about 40% of the 9% decline in the local geomagnetic field strength since 2000.

Repeating the standard FC transport calibration while accounting for the trend in the geomagnetic field renders the trend in FC transport statistically insignificant and reduces the AMOC trend to marginal significance. The new transport estimate is also a better fit to the calibration data as well as independent altimetry data. Unlike the uncorrected FC time series, the calibration data scatter uniformly about the new transport time series, consistent with random, unbiased errors. This makes a strong case that the new FC time series is a better representation of the true transport of the Florida Current than the original time series.

Recommendation

Given the significance of the AMOC and FC transport to the AMOC, this is a very important result that may require some to rethink long-held ideas about the current state and recent history of the AMOC.

- Nature Communications is an appropriate venue to communicate such a result.

- Writing: The manuscript itself is well-written and clear.

- References: The references are thorough, although not encyclopedic. (For example, it does not address a similar argument in the literature about the slowing of the Gulf Stream downstream of Cape Hatteras. However, the two related systems are sufficiently different that such attention

would likely be tangential.)

- Figures: The figures are, by and large, clear and necessary to the manuscript's narrative. My only complaint is that the 1-minute data shown in figure 5 are so dense it is impossible to distinguish anything but the extreme events. Using coarsened data would make the figure more legible while making the same point.

- Methods: The description of the calibration is a little unclear. Equations (2) and (4) make it seem like β could be an arbitrarily complex function of time. However, reviewing the code makes it clear that, within large blocks of time, β is a constant determined by regression over the calibration data. This same regression determines α, which isn't given a time index. A few words to clarify that both of these coefficients are piecewise constants would be helpful. In retrospect, one can figure this out based on lines 482–487, but I had to work backward from the code to understand this passage.

Other than these small issues, I see no reason not to publish this manuscript promptly.

Reviewer #2 (Remarks on code availability):

By "reviewed the code", I mean I've checked to make sure it is accessible (it is) and gave it a brief review to ensure that it made sense. I did not have an opportunity to actually run the code, although it appears clear how one would if one wanted to.

We would like to thank both reviewers for their time and for providing helpful and constructive comments on our manuscript. We have tried to address all concerns raised by the reviewers. Our answers to reviewers' comments are provided below in blue font.

Reviewer #1 (Remarks to the Author):

Major concerns

1. Line 86-89: I believe records from calibration cruises were also adopted in estimating FCT before 1998. Thus, I am wondering why the geomagnetic correction is applied for the period 1982-1998 but not for 2000-present before the work presented in the manuscript?

As we mentioned in the manuscript, the long history of the FC measurements involved several research programs and generations of scientists. When the WBTS began collecting cable data in 2000, there had been a change in the lead organization, the leadership, and the personnel involved in this monitoring program. This transition had also resulted in partial loss of pre-2000 raw voltage data for the reasons we do not know. Because the geomagnetic correction that was applied to the pre-2000 cable record was based on geomagnetic measurements relatively far from the Straits of Florida (Larsen, 1992), it was decided that the high-frequency geomagnetic-induced fluctuations in voltages could be effectively removed by applying a 3-day low-pas filter (Meinen et al., 2010). We have added this explanation in lines 466-474 of the revised manuscript.

Because in the early 2000s the moder era cable record was still short, it was reasonably assumed that the impact of the secular change in the EMF was very small, and it would be minimized by adjusting the offset β in eq. (2). So, the different approach adapted after 2000 was justified at the time, but a problem with this approach is that one needs to monitor the FC for a long time in order to detect meaningful systematic biases between the cable and ship section transports related to the EMF. This is exactly what happened. As the cable record became long enough (23 years) it became clear that a correction for the secular change in the EMF is necessary as the adjustments of the offset are very irregular and they are targeted at minimizing large step-like differences due to system failures. We have added two additional paragraphs in lines 476-493 of the revised manuscript to explain this.

2. The trend in the vertical component of the Earth's Magnetic Field (EMF) is fundamental to the geomagnetic correction and the conclusions of this manuscript. What is the uncertainty of the trend in the vertical component of EMF (F_z)? Is the uncertainty comparable with the trend itself? A detailed discussion on this matter is necessary to establish the reliability of the conclusions.

We thank the reviewer for this important remark. To address it, we have included a new subsection in Materials and Methods: "*Correcting voltage data for the secular change in the EMF*". Please see the first two paragraphs of this subsection. Unfortunately, we do not know the local error in the vertical component of EMF as it is not provided as part of the IGRF model. However, based on relevant publications (Alken et al., 2021; Lowes, 2022), we note that after 2000, when satellite data is available, the RMS vector uncertainty of the EMF is close to 10 nT.

For the vertical component of the EMF this uncertainty is even lower, suggesting that it amounts to about 5% of the local rate of the secular decline in Fz (~150 nT per year).

3. Line 287-289: The Florida Current is not only the surface limb of AMOC, but also the western boundary component of the wind-driven circulations in the subtropical gyre. A Florida Current decline may indicate either a weakened AMOC or a weakened subtropical gyre. Other factors, such as changes in local wind stress or warming coastal water, may also affect FCT. Therefore, a decline in FCT is not necessarily linked to an AMOC slowdown. I recommend the authors add some brief discussions about the source of FC and how to interpret trends in FCT.

We absolutely agree with the reviewer. A decline in the FCT does not necessarily indicate an AMOC slowdown as it could be compensated by the mid-ocean transport (east of the Bahamas). The source of the FC is described in the first paragraph of Introduction. To address the reviewer's recommendation, we have revised the following sentence in the Discussion section (lines 291-294): "Because the FC is the major contributor to the northward transport of both the upper-limb of the AMOC and the subtropical gyre circulation in the North Atlantic, it is reasonable to expect that on sufficiently long time scales a FC decline could indicate a slowdown in the AMOC and/or the gyre circulation."

4.Line 371: The old FCT was estimated from equation (2) without the geomagnetic correction. Differences between the cable observations and ship measurements, including the long-term trend introduced by changes in the geomagnetic field, should be compensated by $\beta(t)$, as mentioned in lines 461-464. With the correction term $\beta(t)$, I expect that the FCT trends derived from cables should be very similar to the trends from calibration cruises. Thus, why are changes in the geomagnetic field not corrected by $\beta(t)$ and the calibration cruises as expected, and the additional geomagnetic correction required?

The reviewer is right that ideally the adjustments of β should (at least partially) compensate for the secular change in the EMF. This thinking is one of the reasons why an explicit correction for Fz has not been applied since 2000. However, in practice, the adjustments of β are not regular. The adjustments were done only after the failures of the recording system, when a systematic bias in transports before and after a system failure could be easily detected. Most adjustments were done in the problematic 2000-2005 time interval. When there are no regular adjustments, it requires a sufficiently long time series to detect a meaningful difference between the cable and ship section transports that could be attributed to the secular change in the EMF. Therefore, we think it is absolutely necessary to explicitly correct voltages for the geomagnetic changes before adjusting β when necessary. We hope that this question is adequately addressed in lines 486-493 of the revised manuscript.

5. Several cables were used to monitor FCT. Are the observed voltages affected by the material or diameter of the cables? Are the FCT, except the geomagnetic correction, derived from the voltages by exactly the same method before and after 2000? How do the differences, if applicable, affect the FCT trend? Since the manuscript is about the long-term trend of FCT, the consistency and differences of how the FCT is derived from cable measurements during different periods are very important and should be documented with more details.

As described in Materials and Methods, two cables have been used between Florida and Grand Bahama Island at about 27N (see lines 349-352). The motion-induced voltages have been measured on center copper conductors (https://atlantic-cable.com/Cables/1972FloridaBahamas/index.htm). We do not think the conductors are different on the two cables. We would like to recall that the 'new' cable started to be used for FCT calculation in 1993, so any changes related to the use of different cables belong to the 1982-1998 record. Unfortunately, we are not able to re-examine this record as the raw voltages for this period have been lost (this is mentioned in lines 304-305).

Minor concerns

1. Line 108-109: The authors mentioned that the decreasing trend of FCT is "largely due to the most recent 2-3 years". I am curious about how much will the geomagnetic correction affect FCT in the most recent 2-3 years?

A decline in the vertical component of the EMF in the Florida Straits accelerated in 1995, but since 2000 this decline has been steady, although not exactly linear (~150 nT per year; see figure below). This means that the effect of EMF in 2021-2023 is not much different from its impact in earlier years.

We have modified the sentence the reviewer is referring to stating that the trend became more significant relative to the noise as the time series length increased not that the trend accelerated in recent years: "The trend became significant relative to the noise as the time series length increased and due to low transports in the most recent 2-3 years of observations now included in the record." (lines 112-114).

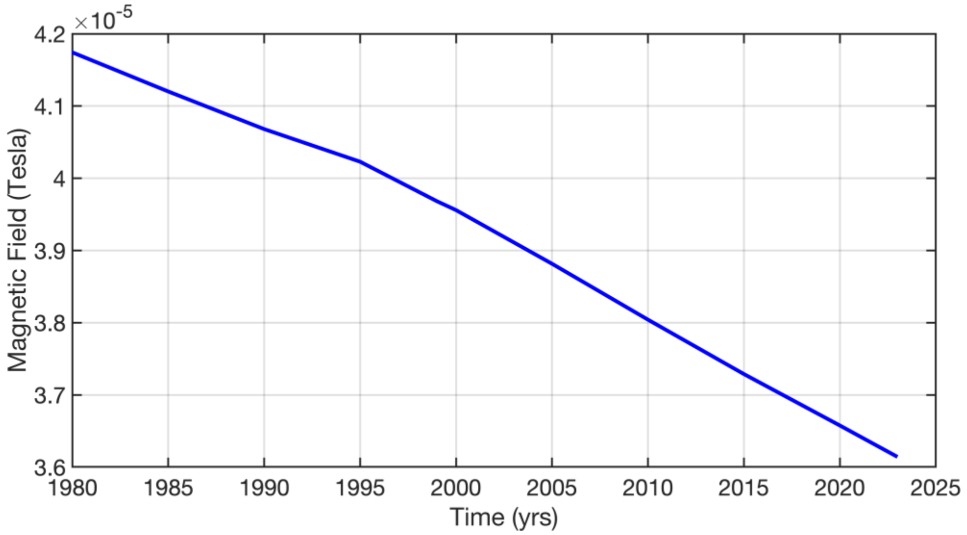

2.In the geomagnetic correction, is it a linear Fz trend adopted for in eq (4)?

The secular change in Fz has been steady since 2000, but not strictly linear. Therefore, in equation (4) we used the yearly estimates of Fz, which account for the nonlinearity. Note, however, that the IGRF model is specified every 5 years (epochs), with linear interpolation used

for intermediate times (see lines 495-501 of the revised manuscript).

3. The water conductivity is affected by its temperature. I am wondering whether the warming trend in Florida Current affected its transport derived from the cables?

As we mentioned in lines 417-423, we calculated the conductivity weighting parameter Cw and found that it is almost identical to the one reported earlier in Larsen and Sanford, 1985. We document that there is a very small and statistically insignificant linear decrease of −0.003 over the 20 years of ship surveys and there is no correlation with the strength of the transport. This means that the impact of ocean conductivity changes on voltages, for example because of the FC warming since 2012, is negligible (lines 421-423).

4. Line 408: “that” -> “than”.

Corrected. Thank you!

Reference

Chen, C., Wang, G., Xie, S.-P., Liu, W., 2019. Why Does Global Warming Weaken the Gulf Stream but Intensify the Kuroshio? J. Clim. 32, 7437–7451. https://doi.org/10.1175/JCLI-D-18-0895.1

We thank reviewer for the reference, which we have included in the Introduction.

Reviewer #1 (Remarks on code availability):

The codes are easy to understand, and I can use them to reproduce the Florida Current transport with geomagnetic correction. The authors also provide cruise observations. With those data, I can reproduce critical results presented in the manuscript.

On the other hand, the code attached is more like the data used in the manuscript with a detailed document. It provides the voltage records and the coefficients to convert voltages into transports, with little information about how those coefficients are created.

To address the last remark made by the reviewer, comments on how the coefficients were obtained have been included in the code in addition to the relevant description in the manuscript (Materials and Methods).

Reviewer #2 (Remarks to the Author):

Recommendation

Given the significance of the AMOC and FC transport to the AMOC, this is a very important

result that may require some to rethink long-held ideas about the current state and recent history of the AMOC.

- Nature Communications is an appropriate venue to communicate such a result.

- Writing: The manuscript itself is well-written and clear.

- References: The references are thorough, although not encyclopedic. (For example, it does not address a similar argument in the literature about the slowing of the Gulf Stream downstream of Cape Hatteras. However, the two related systems are sufficiently different that such attention would likely be tangential.)

We have added a sentence with two additional references (one suggested by reviewer 1) related to the Gulf Stream and its different behavior downstream and upstream of Cape Hatteras (lines 75-78): "*If the AMOC is indeed slowing down on a centennial timescale, its upper branch, primarily composed of the FC and the Gulf Stream in the subtropical North Atlantic, may also be weakening (**Error! Reference source not found.**), although the trends downstream and upstream of Cape Hatteras can be different (**Error! Reference source not found.**).*"

- Figures: The figures are, by and large, clear and necessary to the manuscript's narrative. My only complaint is that the 1-minute data shown in figure 5 are so dense it is impossible to distinguish anything but the extreme events. Using coarsened data would make the figure more legible while making the same point.

As reviewer suggested, we tried plotting subsampled 1-min voltage data, but, in our opinion, the result was not visually better than plotting the original 1-min time series. The whole objective of Fig. 5 is to demonstrate the record-long decline in voltages, which is clearly seen despite the noisiness of the 1-min record. Because of these considerations, we prefer to keep the original figure showing the 1-min record.

- Methods: The description of the calibration is a little unclear. Equations (2) and (4) make it seem like β could be an arbitrarily complex function of time. However, reviewing the code makes it clear that, within large blocks of time, β is a constant determined by regression over the calibration data. This same regression determines α, which isn't given a time index. A few words to clarify that both of these coefficients are piecewise constants would be helpful. In retrospect, one can figure this out based on lines 482–487, but I had to work backward from the code to understand this passage.

We thank the reviewer for this comment, pointing out that the presentation of β in equations (2) and (4) with a time index is not correct and causes confusion. While β has been adjusted several times, the initial β was determined at the same time with α during by regression, and it would be a true constant if the cable recording system were stable. To address this issue in the manuscript, we have edited the equations (2) and (4) by removing the time index from β. We have also substantially modified lines 376-381 in the subsection "Submarine cable measurements" and the subsection "Differences in the cable data processing" (lines 476-485).

REVIEWERS' COMMENTS

Reviewer #1 (Remarks to the Author):

The response of ocean circulations, particularly Atlantic Meridional Overturning Circulation (AMOC) and western boundary currents, to anthropogenic forcing is crucial to understanding mechanisms behind climate change and predicting future climate. Many recent works suggest that FCT has weakened with AMOC in recent decades. Based on in-situ observations, the presented study shows that the Florida Current transport (FCT) has been stable in the past ~40 years, with a false decline trend due to a trend in the Earth's magnetic fields around the Florida Straits. This result is significant and underscores significant uncertainties in our understanding of the current status of the climate system.

The manuscript is well-written and thoroughly referenced, appealing to a diverse audience. All my previous concerns have been addressed in this revised manuscript, and I recommend the manuscript be published in Nature Communications.

I have one minor suggestion regarding the description of beta, which may not require another round of revision: The current description of beta in equations (2) & (4) is confusing. While the manuscript states a constant value of beta (4.2 Sv), it also mentions adjustments "in problematic time intervals" (in the manuscript) and updates approximately once per year during 2001-2014 (in codes). I guess a more straightforward way would be adding a table of beta over time.

Reviewer #1 (Remarks on code availability):

I can reproduce the Florida Current transport with geomagnetic correction easily with the code. The code also provides lots of details about how the Florida Current transport is derived, which would be a useful reference for the community.

Reviewer #2 (Remarks to the Author):

The authors have responded appropriately to all of my original comments. The paper is important and timely. I recommend publication.

Response to reviewers

We would like to thank both reviewers for their time to evaluate our revised manuscript and reviewer 1 for providing an additional suggestion.

Reviewer 1 stated: "*I have one minor suggestion regarding the description of beta, which may not require another round of revision: The current description of beta in equations (2) & (4) is confusing. While the manuscript states a constant value of beta (4.2 Sv), it also mentions adjustments "in problematic time intervals" (in the manuscript) and updates approximately once per year during 2001-2014 (in codes). I guess a more straightforward way would be adding a table of beta over time.*"

We have revised the text in the following manner:

1) We have removed the sentence that followed equation (2): "*The calibration factor α has been retained constant since then, but the offset β has been adjusted in problematic time intervals based on comparisons with ship section transports as described below*." (Lines 379-381 in the previous version of the manuscript). Because a detailed description of the adjustments applied to beta is provided in the section "Differences in the cable data processing", we hope that this way a reader is not confused by adjustments to beta prematurely, and both alpha and beta are considered constant as in the original equation (2) from Larsen (1992).
2) For the same reason as above, the second sentence in the next section "Ship-borne measurements" has been edited as follows: "*Therefore, routine validation of cable-derived transports is required to ensure the stability of the recording equipment and the robustness of the cable record*."
3) A detailed description of why and how beta was adjusted is provided in the second paragraph of the section "Differences in cable data processing". Because beta is a constant in equation (2), in order to make a clearer distinction between beta and an optional adjustment to it, we add the following sentence to the second paragraph: "*This adjustment to β represents a piecewise constant function, different from zero only in problematic time intervals.*" (Lines 477-479 of the revised manuscript).

We hope that these small changes make the methodology section clearer and address the reviewer's concern. We do not think that adding a table with adjustments to beta would be useful for readers interested in detail in addition to what is already included and explained in the code. Furthermore, we are already at the limit of 10 display items (8 figures + 2 tables).
